# Enriched H3K4me3 marks at Pm-0 resistance-related genes prime courgette against *Podosphaera xanthii*

**DOI:** 10.1093/plphys/kiab453

**Published:** 2021-09-21

**Authors:** Theoni Margaritopoulou, Dimosthenis Kizis, Dimitris Kotopoulis, Ioannis E Papadakis, Christos Anagnostopoulos, Eirini Baira, Aikaterini Termentzi, Aikaterini-Eleni Vichou, Carlo Leifert, Emilia Markellou

**Affiliations:** 1 Scientific Directorate of Phytopathology, Benaki Phytopathological Institute, Athens 14561, Greece; 2 Scientific Directorate of Pesticides' Assessment & Phytopharmacy, Benaki Phytopathological Institute, Athens 14561, Greece; 3 Faculty of Crop Science, Agricultural University of Athens, Athens 11855, Greece; 4 SCU Plant Science, Southern Cross University, Lismore, Australia; 5 Department of Nutrition, IMB, University of Oslo, Oslo 0372, Norway

## Abstract

Powdery mildew (PM) disease, caused by the obligate biotrophic fungal pathogen *Podosphaera xanthii*, is the most reported and destructive disease on cultivated Cucurbita species all over the world. Recently, the appearance of highly aggressive *P. xanthii* isolates has led to PM outbreaks even in resistant crops, making disease management a very difficult task. To challenge this, breeders rely on genetic characteristics for PM control. Analysis of commercially available intermediate resistance courgette (*Cucurbita pepo L. var. cylindrica*) varieties using cytological, molecular, and biochemical approaches showed that the plants were under a primed state and induced systemic acquired resistance (SAR) responses, exhibiting enhanced callose production, upregulation of salicylic acid (SA) defense signaling pathway genes, and accumulation of SA and defense metabolites. Additionally, the intermediate resistant varieties showed an altered epigenetic landscape in histone marks that affect transcriptional activation. We demonstrated that courgette plants had enriched H3K4me3 marks on *SA-BINDING PROTEIN 2 and YODA* (*YDA*) genes of the Pm-0 interval introgression, a genomic region that confers resistant to Cucurbits against *P. xanthii*. The open chromatin of *SA-BINDING PROTEIN 2* and *YDA* genes was consistent with genes’ differential expression, induced SA pathway, altered stomata characteristics, and activated SAR responses. These findings demonstrate that the altered epigenetic landscape of the intermediate resistant varieties modulates the activation of *SA-BINDING PROTEIN 2* and *YDA* genes leading to induced gene transcription that primes courgette plants.

## Teaser

Open chromatin of resistance-related genes induces a primed state and systemic acquired resistance responses in courgette varieties against the biotrophic pathogen *Podosphaera xanthii.*

## Introduction

Powdery mildew (PM) fungi are obligate biotrophic plant pathogens that can only grow and reproduce on living host cells (Belanger, 2002) and plants have evolved intricate recognition arrays and defense mechanisms for these pathogens. Pattern recognition receptors located on the plant cell membrane recognize specific pathogen-associated molecular patterns (PAMPs) produced by the pathogens. This identification initiates a cascade of events leading to PAMP-triggered immunity (PTI), a state characterized by extensive transcriptomic reprogramming leading to physiological changes designed to limit pathogen infection (Boller and Felix, 2009; Monaghan and Zipfel, 2012). Specifically, PTI induces defense hormone biosynthesis, expression of defense genes, and physical reinforcement of cell walls through production of callose and lignin in the host cell (Wan et al., 2008; Han et al., 2010). It also leads to the production of Salicylic acid (SA), a major plant hormone that regulates defense responses against biotrophic pathogens (Vlot et al., 2009), and the synthesis of a range of antimicrobial secondary metabolites (Wink, 2018). In response to PTI, pathogens may produce effector proteins into host cells to disrupt PTI. Effector proteins are designed to disturb resistance-related processes and signaling in the host plant and generate effector-triggered susceptibility (Hann and Rathjen, 2010). Plants have evolved a second line of vigorous defense, which may be expressed when speciﬁc intracellular host plant receptors/sensors recognize these race-speciﬁc fungal effectors and/or host proteins that are altered by effector activity. This secondary defense response has been described as effector-triggered immunity (ETI; Collier and Moffett, 2009). Both PTI and ETI are usually followed by systemic activation of defense mechanisms in distant, uninfected plant tissues known as systemic acquired resistance (SAR), providing resistance against subsequent pathogen challenges (Durrant and Dong, 2004; Mishina and Zeier, 2007). SAR includes a primed state of defense-related genes that facilitates faster and stronger responses to imminent pathogen attacks and was recently associated with alterations in epigenetic traits (Jaskiewicz et al., 2011; Ding and Wang, 2015; Hoang et al., 2018).

It is widely accepted that SAR depends on SA signaling and constitutes a long-lasting form of resistance against a broad spectrum of (hemi-)biotrophic pathogens. Plants were shown to have two pathways to synthesize SA: the isochorismate synthase and the phenylalanine ammonia-lyase (PAL) pathways, which both start from chorismate (Lefevere et al., 2020). Some plant genes/regulatory factors associated with SA signaling have also been identified, such as *NONEXPRESSOR OF PATHOGENESIS-RELATED GENE 1/3/4* (*NPR1/3/4*; Cao et al., 1997; Innes, 2018) and the *WRKY* family of transcription factors (Eulgem and Somssich, 2007).

Chromatin structure and function are important for the regulation of gene transcription. Histones, the core proteins of chromatin, are subjected to many posttranslational modifications that are associated with distinct transcription states. Chromatin modifications have recently been described as essential epigenetic mechanisms underlying plant defense against pathogens and potential transcriptional regulators for innate immunity in plants (Ding and Wang, 2015; Alonso et al., 2019). For example, studies of interactions between Arabidopsis (*Arabidopsis thaliana*) and both fungal (*Hyaloperonospora arabidopsidis* the causing agent of downy mildew) and bacterial (*Pseudomonas syringae* pv. *tomato* (Pst) DC3000) plant pathogens, have shown that histone modifications affect/regulate the expression of key defense genes (Xia et al., 2013; Ramirez-Prado et al., 2018). Moreover, models describing the dynamic interplay between covalent chromatin modifications and the activity of SA-responsive genes are available (van den Burg and Takken, 2009).

The obligate biotrophic pathogen *Podosphaera xanthii* (Castagne) U. Braun & Shishkoff, the main causing agent of PM, is the most reported and destructive disease on cultivated Cucurbita species such as cucumber, melon, watermelon, pumpkin, and squash in a global manner (Krístková and Lebeda, 2000; Pérez-García et al., 2009; Pirondi et al., 2015). PM can be easily spotted as a white mycelium on leaves, petioles, and stems of plants. As disease progresses, chlorotic lesions appear on leaf surfaces and plants deteriorate due to photosynthesis inhibition. PM is controlled by regular application of synthetic chemical and/or sulfur-based fungicides. However, the long-term use of fungicides has led to the emergence of *P. xanthii* strains that are resistant or less susceptible to synthetic chemical fungicides making disease control more difficult (Vielba-Fernández et al., 2020).

Traditional plant breeding techniques rely on natural genetic diversity for the selection of desirable agronomic traits. As a result, several commercial varieties and breeding lines of cucumber, melon, and squash with resistance to PM have been released. However, in recent years, the appearance of highly aggressive *P. xanthii* isolates has led to PM outbreaks even in resistant crops, such as watermelon (Pérez-García et al., 2009).

Until today, genetic resistance to PM has only been identified in a few wild accessions of *Cucurbita moschata*, while in the highly susceptible *Cucurbita* *pepo*, there are some accessions that exhibit partial resistance in field and growth chamber experiments. However, this partial resistance is not sufficient for disease control (Holdsworth et al., 2016). Genotyping-by-sequencing mapped the location of the resistance, derived from the introgression of a major resistance gene from the wild species *Cucurbita okeechobeensis* subsp. *martinezii* named Pm-0 gene, to a Pm-0-interval of 76.4 kb containing 14 putative genes (Holdsworth et al., 2016). Sequence analysis of the putative genes revealed homologies to genes of other genera that are known to be involved in disease resistance.

In this study, early responses of inoculated plants with *P. xanthii* conidia were recorded and compared in commercially available susceptible (S) and intermediate resistance (IR) courgette varieties. We detected significant changes in transcript accumulation between varieties especially in noninoculated plants. Most profound was the increased transcript accumulation of the SA signaling pathway genes and the PTI marker *WRKY29*. Microscopic observations revealed differential increase in callose production as well as significant differences of stomata characteristics particularly between S and IR plants in both treatments. Analysis of varieties’ epigenetic background revealed variation on histone marks, that regulate gene expression, between noninoculated and inoculated plants. Moreover, the increased H3K4me3/H3K27me3 ratio in noninoculated IR plants displayed the epigenetic influence of open chromatin state on positive regulation of gene transcription. Detailed examination of two genes of the Pm-0-interval at the chromatin level, *SA-BINDING PROTEIN 2* (*SABP2)* and *YODA* (*YDA)*, revealed enrichment of H3K4me3 marks. Finally, leaf metabolomic profiling demonstrated SA accumulation and increased production of defense metabolites not only after pathogen inoculation but also in noninoculated IR varieties, reinforced the role that Pm-0-interval exerts on resistance mechanisms. Together, our data suggest that *SABP2 and YDA* Pm-0 genes modulate resistance against *P. xanthii* by setting plants in a primed state and that epigenetic background of courgette varieties on genes has an important regulatory function on defense and induced SAR responses that could be further explored for production of varieties with enhanced resistance.

## Results

### IR *C. pepo* varieties exhibit primed callose and defense metabolites production

**Figure 1:**
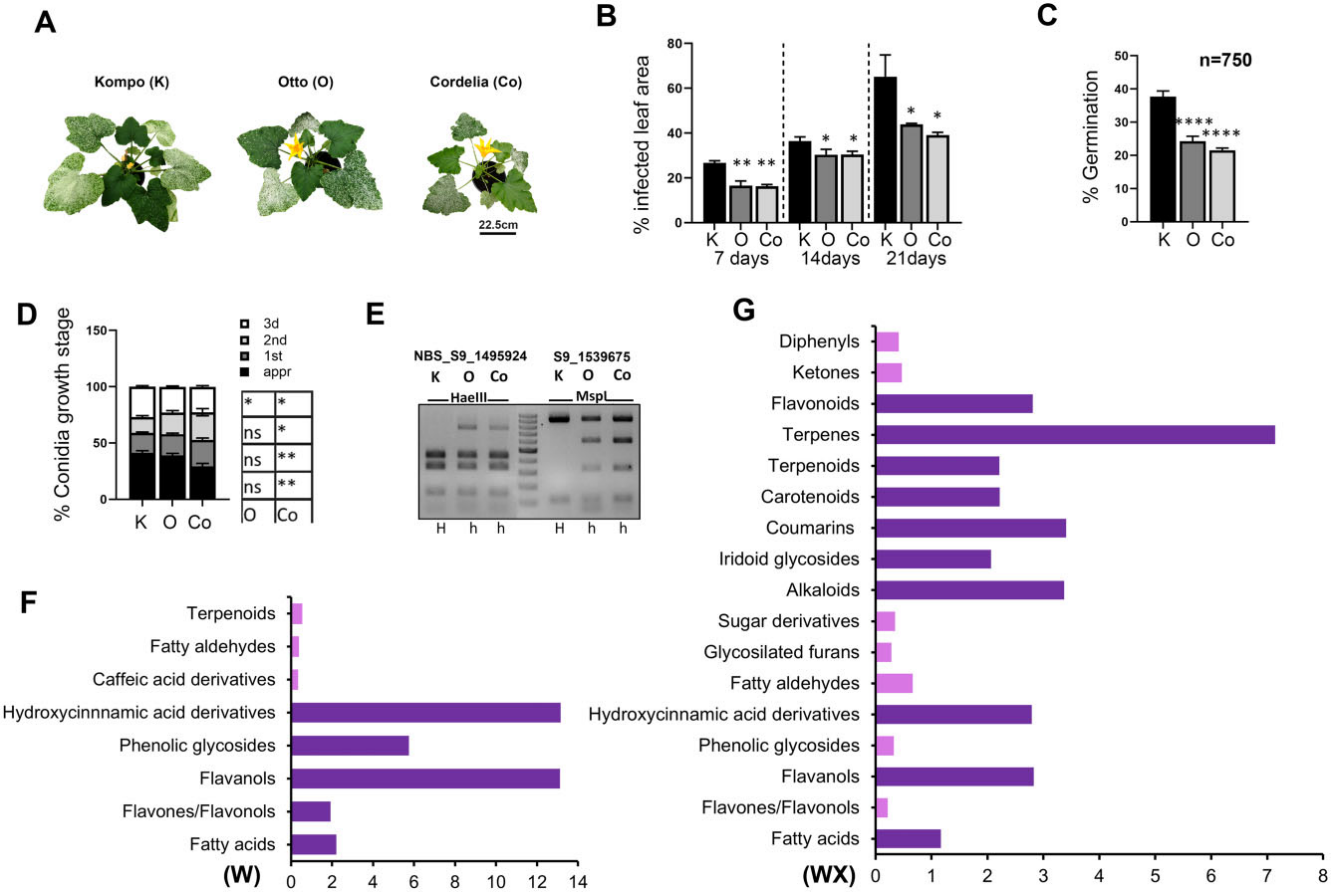
IR varieties show delayed *P. xanthii* infection and accumulation of defense metabolites. A, Disease symptoms of Kompo, Otto, and Cordelia plants. Whole 2-week-old courgette plants of each variety were artificially inoculated with an aqueous conidial suspension of *P. xanthii*, at a titer of 1.5 × 10^5^ CFU mL^−1^. Disease severity was monitored over a period of 3 weeks. Plants were photographed at 14 dpi. B, Disease severity assessments. Three plants from each variety were set in a single plant plot randomized block design with six blocks per replicate. Disease severity was assessed by visual estimation of the percentage infected leaf area. Data represented the mean of three plants ± sem (*n* = 3). Asterisks represent statistically significant differences (*P < 0.05, **P < 0.01) of Otto and Cordelia compared to Kompo, respectively, as determined by one-way ANOVA followed by Dunnett’s posthoc test. C, variety effect on conidia germination. Germination was evaluated at 36 hpi on courgette leaves. At least three leaf areas from three different leaves of each variety were stained with Lactophenol blue and the germinated conidia were counted under the microscope. The total number of germinated conidia was divided into four categories to evaluate the percentage of conidia growth stages on the leaves of each variety. Data represented the mean of three measurements of 250 conidia ± sem (*n* = 3). Asterisks represent statistically significant differences (****P < 0.0001) as determined by one-way ANOVA followed by Dunnett’s posthoc test. D, Conidia growth stages differ between varieties. About 750 conidia on leaves from each variety were measured. Table shows the comparisons of each growth stage between varieties. Asterisks represent statistically significant differences (*P < 0.05, **P < 0.01, ns, not significant) as determined by one-way ANOVA followed by Dunnett’s posthoc test. appr: appressorium formation, 1st: one primary hypha, 2nd: two primary hyphae, 3rd: three primary hyphae. Error bars to each growth stage in each variety. E, Restriction enzyme digests with *Hae*III and *Msp*I endonucleases of the PCR products that were amplified with primers for the Pm-0-interval markers NBS_S9_1495924 and S9_1539675, respectively. H represents homozygous and h the heterozygous variety. F and G, Whole untargeted metabolome profiling was performed for noninoculated (F) and inoculated (G) plants. For comparison reasons, the outcome of the S analysis was compared to that of both IR varieties whose data were pooled. Data represent the ratio between pooled Otto/Cordelia results to Kompo results on the identified metabolite classes. IRs: Otto/Cordelia, S: Kompo, W: noninoculated, WX: inoculated with 1.5 × 10^5^ CFU mL^−1^*P. xanthii* conidia. Purple color indicates upregulation and pink indicates downregulation of the detected classes.

In order to examine the relationship between variety effect and PM disease progress, one susceptible (S, Kompo) and two intermediate resistant (IR, Otto, Cordelia) varieties were selected. Our disease severity assay showed that IR plants were able to delay fungal infection for up to 21-d postinoculation (dpi) when compared to S (Figure 1, A and B; Supplemental Figure S1). Typically, at 24 hours post inoculation (hpi), *Erysiphe cichoracearum* (a PM species closely related to *P. xanthii*) conidia germinate, form appressoria and haustoria, and by 2 dpi three elongated fungal hyphae are formed from a single conidium on a susceptible Arabidopsis variety (Guo et al., 2013). WGA-488 and Lactophenol blue staining of *P. xanthii* conidia on courgette leaves gave similar results at 36 hpi (Supplemental Figure S2), but there were significant differences in fungal infection between varieties. Specifically, we observed a significant reduction (10%–15%) of conidia germination on leaves of the IR plants when compared to those of the S variety at 12 and 36 hpi (Figure 1C; Supplemental Figure S1C). Also, at 36 hpi, we found significantly fewer (10%) conidia with appressorium formation in Cordelia, compared to Kompo leaves, and the proportion of conidia that developed three primary hyphae was significantly (4%) lower in Otto and Cordelia when compared to Kompo. In Cordelia, both the first stage of appressorium formation and the later stage of hyphal growth were found to be significantly delayed compared to Kompo leaves (Figure 1D). Recent studies in squash varieties have revealed that Pm-0-interval introgression from the wild *C. okeechobeensis* subsp. *martinezii* variety is conferring resistance against *P. xanthii* infection (Holdsworth et al., 2016). To test the presence of Pm-0 introgression in our courgette varieties, marker-assisted genotyping was performed according to Holdsworth et al. (2016) who developed the markers we used. The generated fragments of the restriction enzyme digestion analysis showed that Kompo is homozygous for the susceptible allele while the two IR varieties are heterozygous for the *C. okeechobeensis* subsp. *martinezii*-derived resistance allele (Figure 1E).

Additionally, global untargeted metabolomic profiling displayed accumulation of secondary defense-related metabolites in the IR varieties. Hydroxycinnamic acid derivatives (HCAs), fatty acids, and compounds of the flavonoid metabolism (Figure 1, F and G; Supplemental Figure S3; Supplemental Table S1 and S2), that are associated with the SA pathway in a positive manner (Zacarés et al., 2007; Kachroo and Kachroo, 2009; Gondor et al., 2016; Stringlis et al., 2019), were detected both before and after pathogen inoculation. Among the annotated metabolites, significant accumulation in the IR plants was detected for the flavonol quercetin (Supplemental Tables S1 and S2), an abundant antioxidant that has been shown to inhibit *Fusurium culmorum* growth in barley (Skadhauge et al., 1997) and recently was shown to induce resistance in Arabidopsis against DC3000 by participating in the SA pathway (Jia et al., 2010). Our results suggest that there could be a correlation between the accumulated SA levels and the biosynthesis of these defense metabolites in the IR plants.

**Figure 2:**
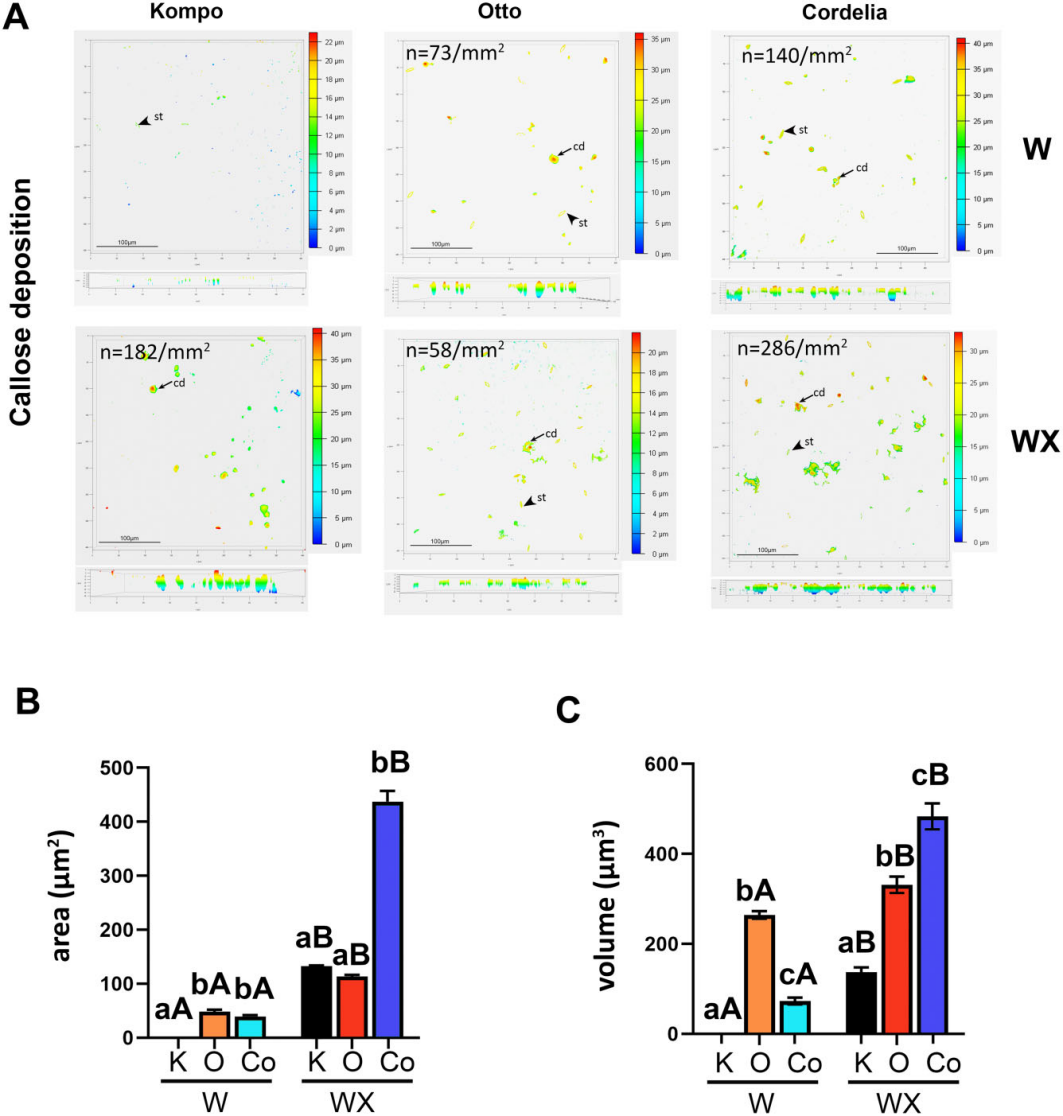
IR leaves embody enhanced callose deposition before and after *P. xanthii* inoculation. A, Aniline blue-stained callose depositions in courgette leaves (one susceptible and two intermediate resistant varieties) before and 36 hpi with *P. xanthii* conidia suspension. Examples of callose deposition (cd) are marked with arrows, while background stomata (st) detection is marked with arrowheads. Pictures are *z*-stacks of optical sections. Color scale bar shows the depth size of the *z*-stack in micrometer. n in each image shows the average number of callose granules on leaves of each variety per square millimeter that were counted with Volocity software (Quorum Technologies Lewes, UK). B and C, Area (B) and volume (C) quantification of callose granules on leaves of all varieties before (W) and 36 hpi (WX) with *P. xanthii* conidia suspension. Approximately 1,000 granules were measured on the leaves of each variety. On Kompo (K) leaves no presence of callose granules was detected before *P. xanthii* inoculation (KompoW samples). Significant differences of variety and inoculation effect were determined with two-way ANOVA following Tukey’s multiple comparisons test (*P* < 0.05). Means of different varieties that received the same inoculation treatments are indicated with lower case letters and *P. xanthii* inoculated and noninoculated plants of the same variety are indicated with different capital letter. Area and volume quantifications were calculated using Volocity software (Quorum Technologies). O: Otto, Co: Cordelia, W: noninoculated, WX: inoculated with 1.5 × 10^5^ CFU mL^−1^*P. xanthii* conidia.

Callose, a (1,3)-β-glucan polymer that is a physiological response during pathogen infection, is deposited in cell walls to increase penetration resistance against fungal attack (Dong and Weng, 2013; Lawrence et al., 2016). No callose was detected in S noninoculated leaves, while the IR varieties showed significant levels of callose formation on noninoculated leaves (Figure 2, A–C; Supplemental Figure S4). Detailed examination of the formed callose granules also revealed differences between the two IR varieties. Noninoculated Otto leaves had lower in number but larger in-depth granules, compared to noninoculated Cordelia (Figure 2, A and C). After *P. xanthii* inoculation, Cordelia showed enhanced production of callose granules, and increased granule size in diameter and depth compared to water-treated plants (Figure 2, A–C). Callose is typically linked with SAR responses when biotrophic pathogens attack plants (Dzhavakhiya et al., 2007), and our observations suggest that the IR plants are in a primed state for imminent pathogen attack.

### SA defense pathway is upregulated in the IR varieties

**Figure 3:**
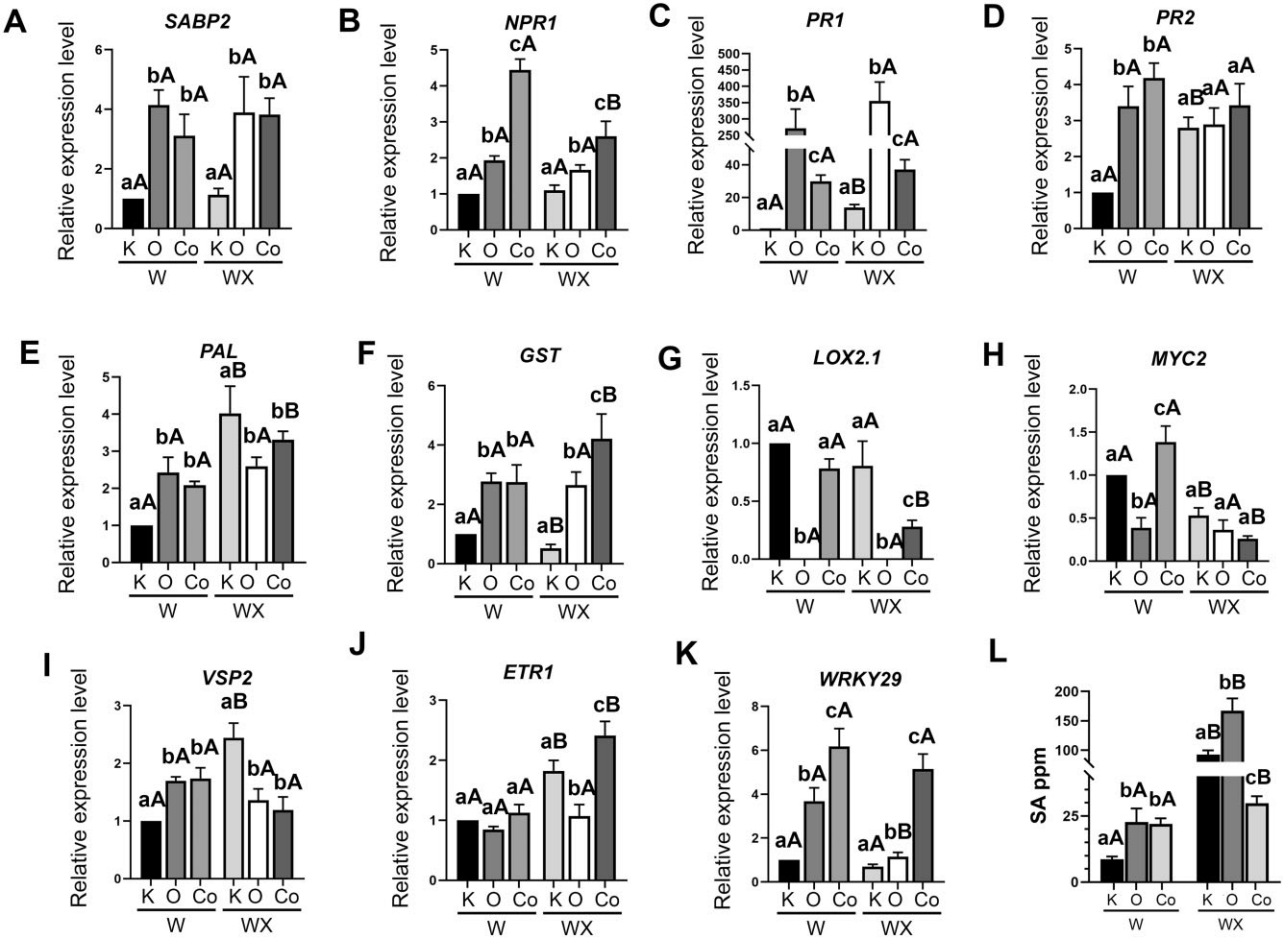
SA-related gene transcriptional upregulation and SA levels accumulation in water treated and *P. xanthii* inoculated leaves of IR courgette plants. A–K, Relative transcript abundance of *SABP2*, *NPR1*, *PR1*, *PR2*, *PAL*, *LOX2.1*, *MYC2*, *ETR1*, *VSP2*, *GST*, and *WRKY29* in leaves of Kompo, Otto, and Cordelia varieties before (W) and after *P. xanthii* inoculation (WX). Transcript abundance was determined by RT-qPCR and normalized to *EFL1a* using the ΔΔ_CT_ method. Control plants were water treated and infected plants were inoculated with 1.5 × 10^5^ CFU mL^−1^ conidia suspension. The third and fourth leaf from the top of each plant was collected 36 hpi and pooled. The relative fold changes were calculated in reference to water treated Kompo leaves. Data are represented as mean ± sem of biological triplicates (*n* = 3), each biological replicate was calculated by averaging two technical replicates. Significant differences were determined with two-way ANOVA following Tukey’s multiple comparisons test (*P* < 0.05). Means of different varieties that received the same inoculation treatments are indicated with lower case letters and *P. xanthii* inoculated and noninoculated plants of the same variety are indicated with different capital letter. L, SA levels were measured in plants noninoculated (W) or inoculated with 1.5 × 10^5^ CFU mL^−1^*P. xanthii* conidia (WX). Statistical analysis was performed as above. K, Kompo; O, Otto; Co, Cordelia.

The Pm-0-interval contains 14 putative genes, with many of them homologous to genes known to be involved in disease resistance in other genera. Among Pm-0 genes, *SABP2* was selected for further examination since it is the only gene shown to be directly implicated in the SA defense signaling against biotrophic pathogens (Supplemental Table S3). *SABP2*, a gene with methyl salicylate (MeSA) esterase activity, is required for SA release from MeSA. *SABP2* is known to be autoregulated by a negative feedback loop and is serving as local and long-distance SAR signal (Vlot et al., 2008; Pokotylo et al., 2019). In this study, reverse transcription-quantitative PCR (RT-qPCR) expression analysis for *SABP*2 revealed significant three-fold transcript difference in the IR leaves compared to S, before and after *P. xanthii* inoculation (Figure 3A).

The SA pathway is a principal component that regulates plant’s defense responses against biotrophic pathogens and our results suggest that *SABP2* participates in SA pathway activation. To evaluate the molecular basis of *SABP2* engagement in plant’s defense mechanisms against *P. xanthii*, the expression of various defense-related genes was examined under noninoculated and *P. xanthii* inoculated conditions by RT-qPCR. The selected genes include the SA-mediated signaling defense pathway genes, *NPR1*, *PATHOGENESIS RELATED 1, 2* (*PR1*, *PR2*) and *PAL*, the SA responsive gene *GLUTATHIONE S TRANSFERASE* (*GST*), the JA-mediated signaling defense genes *LYPOXYGENASE 2.1* (*LOX2.1*), *VEGETATIVE STORAGE PROTEIN 2* (*VSP2*) and *JASMONATE INSENSITIVE* 1 (*MYC2*), the Ethylene-mediated signaling defense pathway gene *ETHYLENE RECEPTOR 1* (*ETR1*), and the PTI marker gene *WRKY29*.

All varieties displayed differences in gene expression before and after *P. xanthii* inoculation. *NPR1* transcript abundance was significantly higher in Otto (two-fold) and Cordelia (4.5-fold) compared to Kompo in noninoculated leaves (Figure 3B). The same pattern of expression was maintained after inoculation, however, with a reduction in IR transcript abundance compared to noninoculated plants. Regarding *PR1* expression, Cordelia demonstrated significant 30-fold gene expression before and 46-fold upregulation after inoculation, while in S plants *PR1* was increased 17-fold after pathogen inoculation (Figure 3C). Otto showed a profound 270- to 350-fold transcript accumulation in both treatments as it was already observed in our previous experiments (Margaritopoulou et al., 2020). *PR2* transcripts were similarly accumulated (3.3- to 4-fold) in the IR varieties in both treatments, while S plants showed a 2.8-fold increase after inoculation (Figure 3D). *PAL* gene expression showed a similar pattern as *PR2* (Figure 3E). *GST* transcript accumulation was increased almost three-fold in IR noninoculated leaves compared to S, with Cordelia showing a stronger upregulation of gene expression (4.5-fold) after *P. xanthii* inoculation (Figure 3F). Even though there was observed variation in gene expression between the IR varieties, our results indicate that the SA defense pathway is induced in the noninoculated IRs that tend to be maintained after *P. xanthii* inoculation.

Measurement of endogenous SA levels revealed higher concentrations in noninoculated leaves of the two IR varieties than Kompo (Figure 3L). Interestingly, SA concentration after pathogen inoculation demonstrated varying pattern among varieties. In S leaves, *P. xanthii* inoculation, as expected, increased endogenous SA levels probably activating SAR in response to pathogen attack. A similar response was observed in Otto leaves, but SA levels increased to a substantially higher level than in Kompo. Unexpectedly, Cordelia leaves showed a modest increase in SA concentration after *P. xanthii* infection, much lower from both Otto and Kompo responses. An explanation could be that SA was more rapidly converted to MeSA for stronger and faster response against *P. xanthii*. This hypothesis is supported by the high transcript levels that were detected in *GST* (Figure 3F). *GST* expression is known to be positively associated and strongly induced by SA (Gullner *et al.*, 2018), suggesting that Cordelia plants produce higher levels of SA but may not accumulate in the leaves to the same extent as in the other two varieties. However, further work is required to test this hypothesis.

On the other hand, the JA signaling pathway genes, *LOX2.1*, *MYC2*, and *VSP2* showed varying transcript accumulation in the noninoculated IR leaves, while after pathogen inoculation their expression was found to be either reduced or not affected (Figure 3, G–I). Interestingly, there were no *LOX2.1* transcripts detected in Otto before and after pathogen inoculation. The JA-mediated signaling pathway is known to interact in an antagonistic manner with the SA-mediated signaling pathway for the regulation of defense responses against pathogen attack (Caarls et al., 2015). The SA–JA cross-talk is a powerful mechanism prioritizing one pathway over the other, depending on the type of the pathogen that attacks the plant with the SA pathway being recognized as triggering resistance to biotrophs while the JA-dependent pathway regulates defense against insect herbivores or necrotrophs (Pieterse et al., 2012). The reduced or nonaffected transcript accumulation of *MYC2*, *LOX2.*1, and *VSP2* in our study, therefore, corroborates this since the induced SA pathway activity in the IR varieties was also associated with lower expression of JA pathway marker genes.

Examination of the *ETR1* transcript accumulation did not show any significant differences in the noninoculated treatment, whereas after pathogen inoculation we noticed significant transcriptional induction in Kompo and Cordelia varieties (Figure 3J), a result that is in agreement with findings showing that ethylene enhances SA-mediated defense responses (Pieterse et al., 2012).

To further investigate the relationship of SA-induced defense pathway with defense responses, the PTI marker gene *WRKY29* (Baum et al., 2019) was studied at transcriptional level. *WRKY29* expression was highly accumulated in noninoculated IR leaves. Specifically, *WRKY29* transcripts abundance was 3.8-fold higher in Otto and 6.2-fold higher in Cordelia compared to Kompo in noninoculated leaves (Figure 3K). After pathogen inoculation, transcript abundance decreased in all varieties, but the relative differences in expression between them remained the same. The results of *WRKY29* gene expression suggest that the components of the PTI response are constitutively primed in the noninoculated IR plants (and particular in Cordelia), similar to Arabidopsis mutants with permanently enhanced immunity to pathogens (Jaskiewicz et al., 2011).

### The IR *C.pepo* varieties are associated with defense response-related reshaped transcriptome

**Figure 4:**
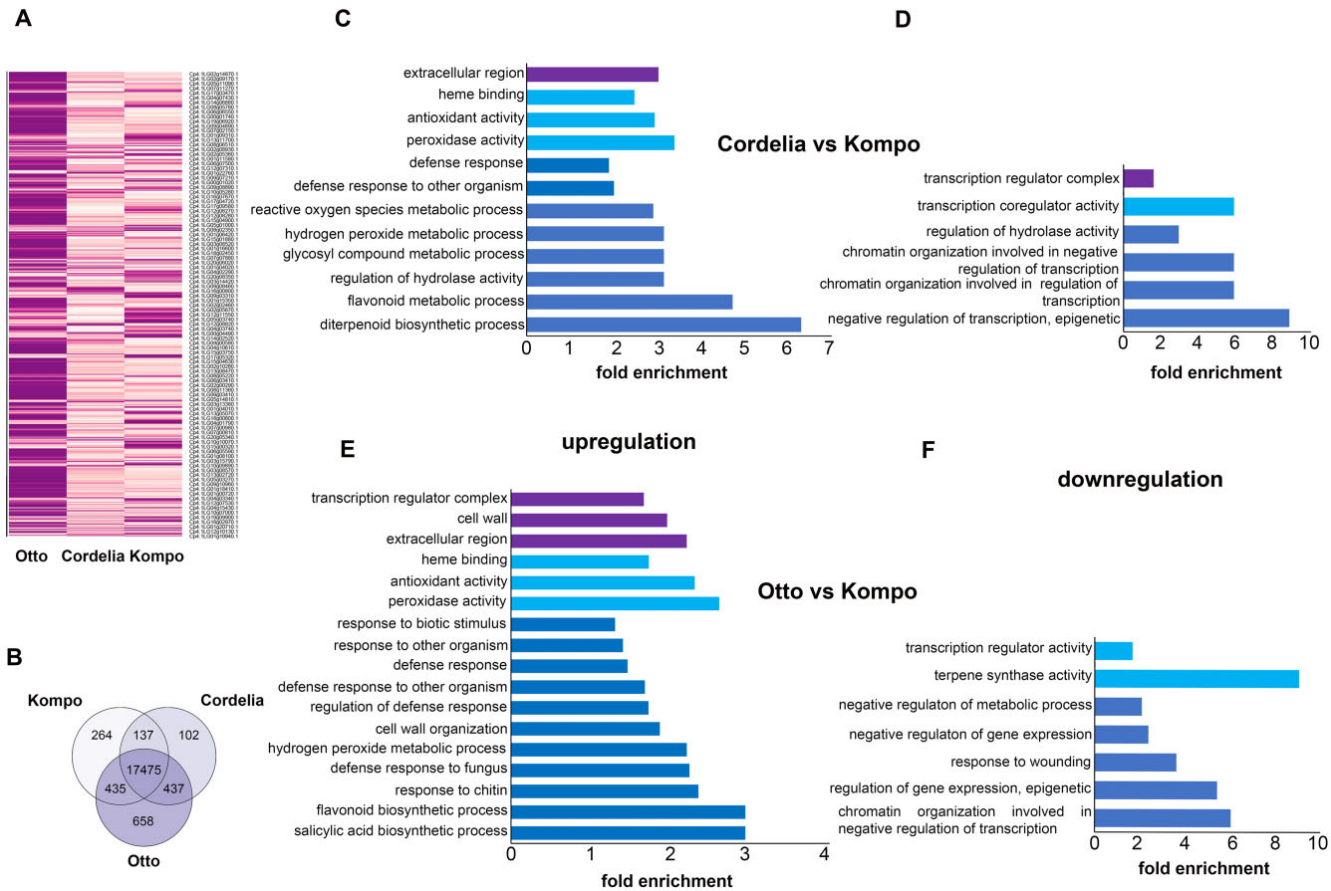
Transcriptional differentiation between the Kompo, Cordelia, and Otto genotypes. A, Heatmap of the identified DEGs in the three genotypes. Lowest and highest expression values for each gene have been normalized between 0 and 1. B, Venn diagram showing overlap of genes differentially expressed in the three genotypes. C–F Enrichment of GO terms in genes that are highly upregulated (C and E) or downregulated (D and F) in the Cordelia vs Kompo and Otto versus Kompo comparison. *P* < 0.05 according to TopGO.

Principal component analysis of all transcriptomes revealed that there is a clear difference in the expression profile of Kompo variety with Cordelia or Otto IR variety, respectively, and inside the IR varieties also (Supplemental Figure S5A). In the Kompo versus Cordelia comparison, out of the 27,868 annotated genes, 23,841 genes were detected, and we obtained 1,514 upregulated and 1,433 downregulated differentially expressed genes (DEGs), while in the Kompo versus Otto comparison out of the 24,108 detected genes we obtained 3,012 upregulated and 2,924 downregulated DEGs (Figure 4, A and B; Supplemental Data Sets S1–S4). Between the IR varieties, out of the 23,978 detected genes, we obtained 1,731 upregulated and 1,971 downregulated DEGs. Our RNAseq data were validated by RT-qPCRs that were already performed and found that the transcriptome data were in good agreement with the RT-qPCR data (Supplemental Figure S5B). In both comparisons of Cordelia and Otto IR variety to Kompo sensitive variety, was revealed significant enrichment for gene ontologies (GOs) related to defense responses for the upregulated genes. In Cordelia variety, we detected significantly enriched GOs for defense response, peroxidase activity, antioxidant activity, and specific GOs to flavonoids and terpenoids defense metabolites (Figure 4D; Supplemental Data Set S5). Interestingly, the detection of the enriched GO of glycosyl compound metabolic process could be associated with the upregulation of the phenylpropanoid pathway that involves PAL gene and HCAs metabolites (Le Roy et al., 2016), reinforcing the connection of the Cordelia variety with the SA defense pathway (Figure 4C). In Otto variety, the GO term enrichment reflected the higher upregulation in gene expression that was detected, compared to the other varieties (Figure 4A). We detected significant enrichment in GOs related to SA biosynthetic process, defense responses, peroxidase activity, cell wall organization, antioxidant activity, and flavonoid defense metabolite biosynthesis. Moreover, we detected specific GOs related to fungal infection, such as defense response to fungus and response to chitin (Figure 4E; Supplemental Data Set S5).

**Figure 5:**
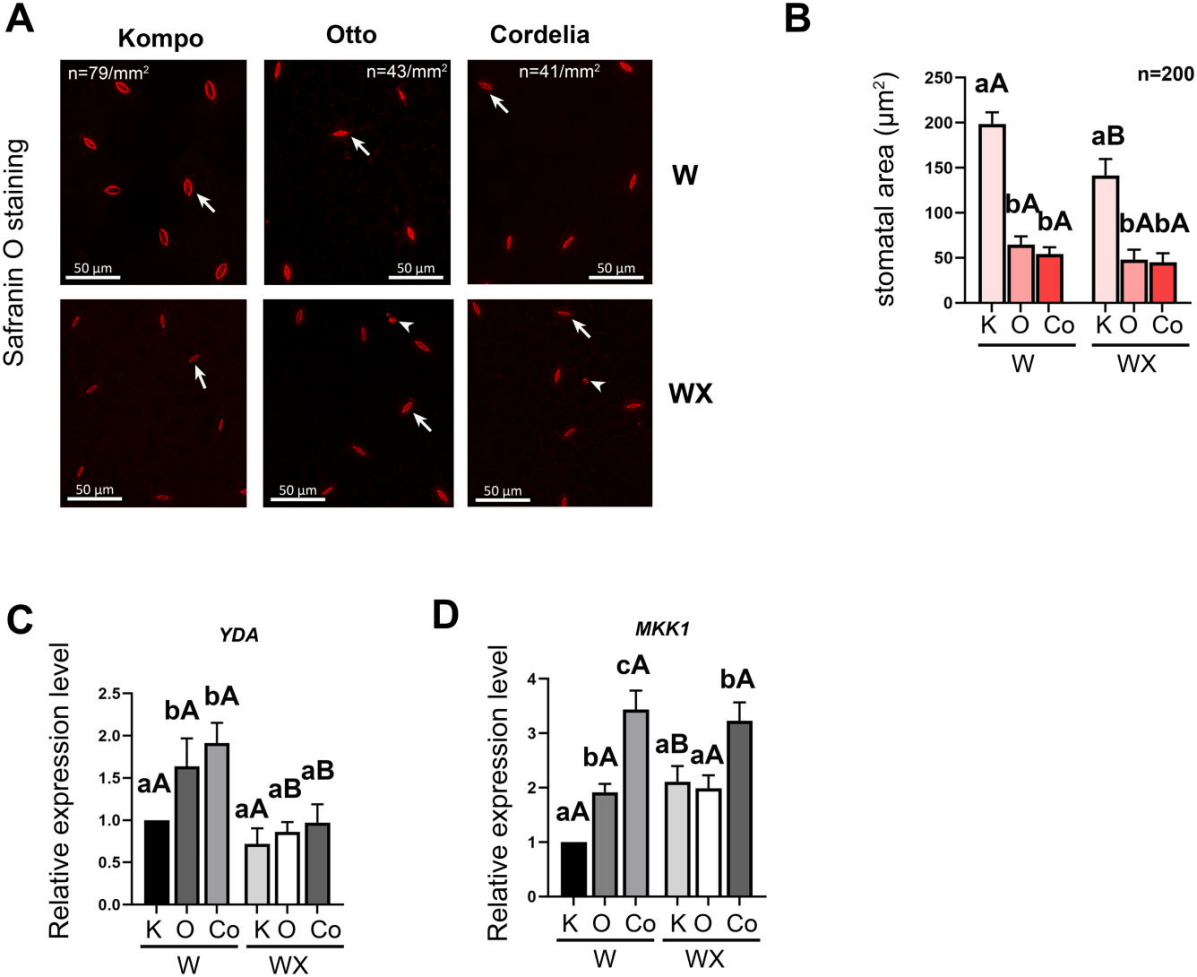
IR leaves have altered stomata traits. A and B, Quantification of stomata density (A) and area (B) in courgette leaves stained with Safranin O before (W) and 36 hpi (WX) with *P. xanthii* conidia suspension. Arrows indicate stomata and arrowheads indicate callose granules. Density and area quantifications were measured using Volocity software (Quorum Technologies). In (B), data are represented as mean ± sem of biological triplicates (*n *= 3). Significant differences in (B) were determined with two-way ANOVA following Tukey’s multiple comparisons test (*P *< 0.05). Means of different varieties that received the same inoculation treatments are indicated with lower case letters and *P. xanthii* inoculated and noninoculated plants of the same variety are indicated with different capital letter. C and D, Relative transcript abundance of *YDA* (C) and *MKK1* (D) was determined by RT-qPCR and normalized to *EFL1a* using the ΔΔ_CT_ method. Control plants were water treated (W) and infected plants (WX) were inoculated with 1.5 × 10^5^ CFU mL^−1^ conidia suspension. Data are represented as mean ± sem of biological triplicates (*n* = 3), each biological replicate was calculated by averaging two technical replicates. Significant differences were determined as above. K, Kompo; O, Otto; Co, Cordelia.

Commonly downregulated genes in both Cordelia and Otto varieties were enriched for GOs related to transcription regulation, chromatin organization involved in negative regulation of transcription, and epigenetic regulation of transcription, indicating an altered mode of transcription in the IR varieties compared to S Kompo variety (Figure 4, D and F; Supplemental Data Set S6).

### Upregulation of YDA Pm-0 gene is related with reinforced leaf physiology

Another Pm-0-interval gene that we studied was YDA.*YDA*, is a mitogen-activated protein kinase (MAPK) kinase that regulates stomatal development and patterning and is described to have a key role in defense mechanisms in Arabidopsis against PM (Bergmann et al., 2004; Sopeña-Torres et al., 2018). Even though stomata are not the main access point of *P. xanthii* for host penetration, there is emerging evidence that stomata density and function play significant role in PM disease progress (Sánchez-Martín et al., 2016; Khan and Rizvi, 2020). Stomata are the main channel of communication of plants with the environment and their density and pattern are determined by both genetic and environmental/climatic factors (e.g. leaf wetness periods, humidity, and temperature) that are also shown to affect the virulence of plant pathogens and host–parasite relationships (Khan and Rizvi, 2020; Samakovli et al., 2020). We, therefore, compared leaf stomatal characteristics in S and IR varieties to investigate whether contrasting patterns of gene expression and PM resistance are associated with differences in leaf stomata architecture and density. Stomatal density on leaves was 2 times higher and the size of stomata 3 times larger in the S Kompo than the IR varieties (Figure 5, A and B). Calculation of stomata index showed that there was a decrease of almost 6% between the IRs and the S variety (10.8%, 12.3%, and 17.2% for Cordelia, Otto and Kompo variety, respectively). Additionally, *P. xanthii* inoculation reduced the stomatal area in Kompo leaves, while in both Otto and Cordelia, stomata covered significantly smaller area that was not affected by pathogen inoculation. *YDA* expression analysis revealed significant *YDA* transcript accumulation in the IR noninoculated leaves compared to S, while *P. xanthii* inoculation downregulated *YDA* expression in the IR leaves to similar transcript levels with S (Figure 5C).

**Figure 6:**
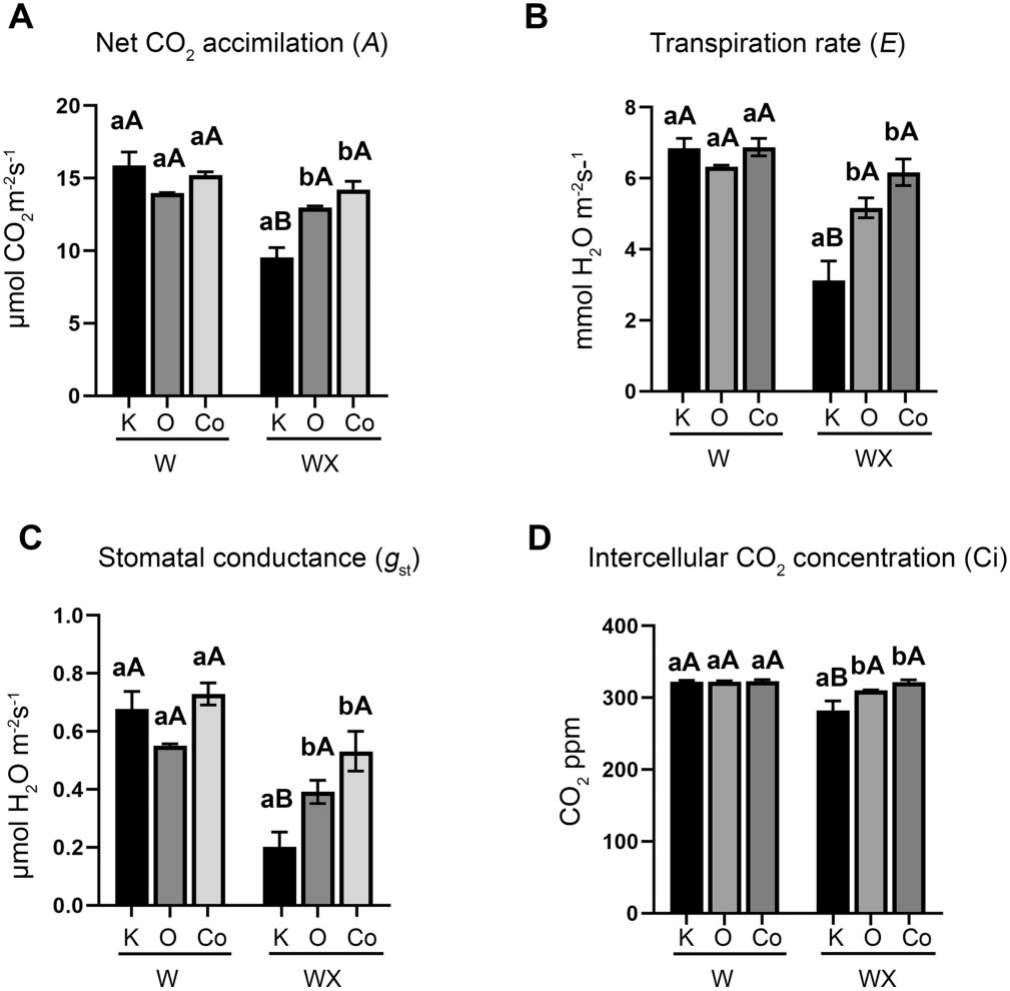
Gas exchange parameters are constant in IR leaves. Li-Cor Li-6400XT measurements of (A) net *A*, (B) *E*, (C) *g_st_* and (D) Ci in courgette leaves before (W) and 36 hpi (WX) with *P. xanthii* conidia suspension. At least two measurements of the third and fourth leaf from the top of each plant were taken. Data represent the mean of four measurements from three biological replicates ± sem (*n* = 3). Significant differences of the variety and inoculation effect were determined with two-way ANOVA following Tukey’s multiple comparisons test (*P* < 0.05). Means of different varieties that received the same inoculation treatments are indicated with lower case letters and *P. xanthii* inoculated and noninoculated plants of the same variety are indicated with different capital letter. K, Kompo; O, Otto; Co, Cordelia; W, water treated; WX, inoculated with 1.5 × 10^5^ CFU mL^−1^ conidia suspension.

To further investigate the association between stomatal pattering and pathogen resistance, transcript levels of the *MITOGEN-ACTIVATED PROTEIN KINASE HOMOLOG* (*MMK1*) were measured. This gene is related to Arabidopsis *MPK6*, which was shown to participate in both ETI and PTI responses. *MPK6* is known to be related to SA defense pathway and is well studied in the YDA–MKK4/5–MPK3/6 module that regulates stomatal development and patterning (Bergmann et al., 2004; Menke et al., 2004; Beckers et al., 2009). Both IRs showed significant *MMK1* transcript accumulation in noninoculated leaves, compared to S, and the observed expression pattern was maintained after pathogen inoculation (Figure 5D). *MMK1* expression was upregulated in Kompo leaves after pathogen inoculation reaching similar transcript levels to Otto expression pattern.

Gas exchange parameters are known to be affected by pathogen attack and plant’s resistance response to pathogens (Voigt and Somerville, 2009). We, therefore, assessed whether the differences in stomata density and morphology between varieties and/or following *P. xanthii* inoculation were associated with changes in gas exchange parameters that were previously linked to contrasting levels of disease resistance. Four essential parameters were analyzed in the S and IR plants, before pathogen inoculation and 3 dpi, revealing significant physiological differences between varieties (Figure 6, A–D). Specifically, net CO_2_ assimilation (*A*) in Kompo leaves significantly decreased from 16 to 9 μmol CO_2_ m_2_ s^−1^ while it remained unchanged in Cordelia and Otto leaves (Figure 6A). Gas exchange parameters (transpiration rate [E], stomatal conductance [*g*_st_], and intercellular CO_2_ concentration (Ci)] significantly decreased in Kompo following pathogen inoculation but remained at similar levels in both IR varieties (Figure 6, B–D). The ratio of net CO_2_ assimilation to water use, known as water use efficiency (WUE), and the ratio of net CO_2_ assimilation to *g*_st_, known as intrinsic WUE, increased in Kompo leaves, but remained at similar levels in Otto and Cordelia leaves after pathogen inoculation (Supplemental Figure S6). This indicates that after pathogen inoculation, water availability decreased, and CO_2_ uptake increased in Kompo leaves, which is consistent with previously reported impacts of *P. xanthii* infection in susceptible plants (Grimmer *et al.*, 2012). On the other hand, the continuous steady water use condition in the IR leaves could imply that the plants did not experience or sense the same level of stress from pathogen infection.

**Figure 7:**
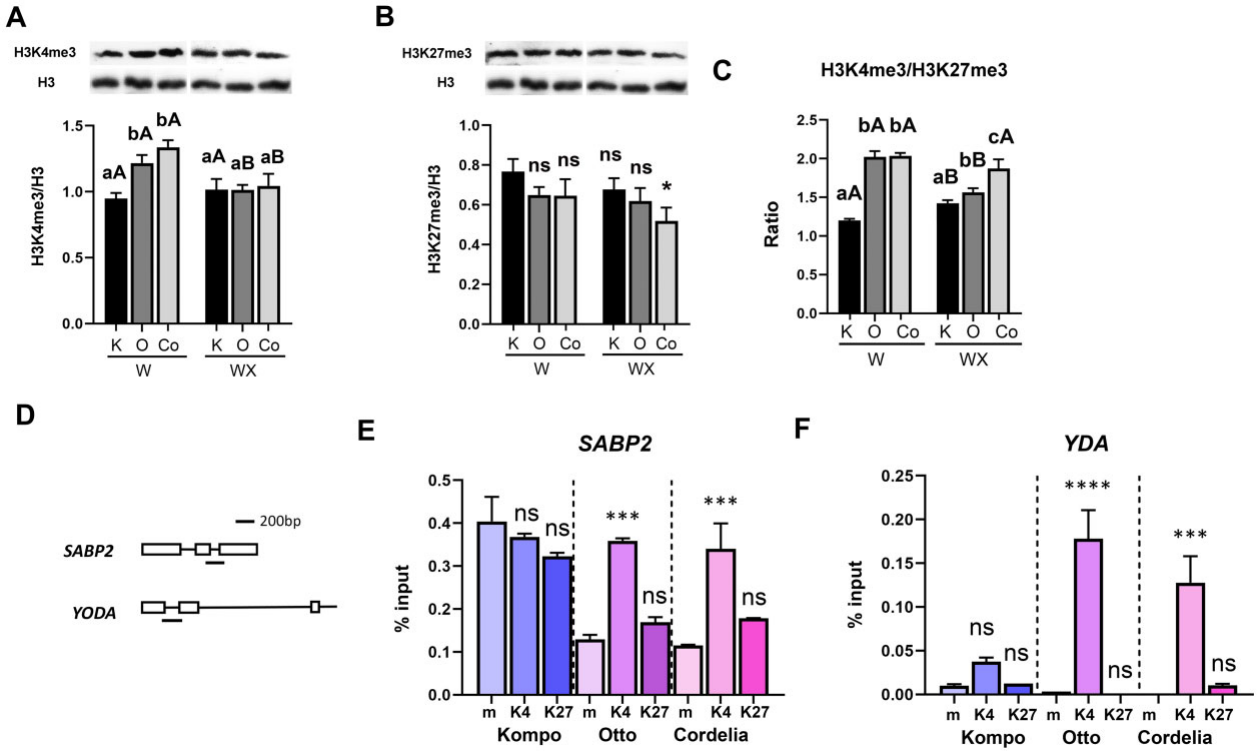
Histone modifications marks are differentially deposited on *SABP2 and YDA* Pm-0 genes in courgette IR varieties. Western blot analysis detecting accumulation of (A) H3K4me3 and (B) H3K27me3 histone modifications. Histone H3 western blot served as internal control of histone modification levels. The same control blot was used for each type of histone modification measured. Below each western blot image is a chart depicting quantification of chemiluminescence signal. Each bar represents mean ± sem of biological triplicates (*n* = 3), each biological replicate was calculated by averaging two technical replicates. In (A), significant differences were determined with two-way ANOVA following Tukey’s multiple comparisons test (*P* < 0.05). Means of different varieties that received the same inoculation treatments, indicated with lower case letters and *P. xanthii* inoculated and noninoculated plants of the same variety indicated with different capital letter, are significantly different according to Tukey’s multiple comparisons test (*P* < 0.05). Asterisk in (B) represents statistically significant difference (*P < 0.01) as determined by one-way ANOVA followed by Dunnett’s posthoc test. Comparison was performed to KW sample. C, Ratio of the relative optical densities of H3K4me3 and H3K27me3 western blots. Statistical analysis was performed as above. D, Schematic representation of the *SABP2 and YDA* gene regions that were tested using qPCR. Boxes indicate exons, first box of each gene represents the first exon. E and F, H3K4me3 and H3K27me3 enrichment at designated loci relative to percent input was determined using ChIP-qPCR in Kompo, Otto, and Cordelia leaves. Asterisks represent statistically significant differences (***P < 0.001, ****P < 0.0001) as determined by one-way ANOVA followed by Dunnett’s posthoc test. Comparison was performed to the m sample in each variety. m represents control ChIP samples (no antibody), K4 represents a-H3K4me3 ChIP samples, K27 represents a-H3K27me3 ChIP samples. K, Kompo, O; Otto, Co, Cordelia; W, water treated; WX, inoculated with 1.5 × 105 CFU mL-L conidia suspension.

### SABP2 and YDA are regulated by H3K4me3 residue enrichment

It is well known that histone surface modifications are key components in the complex and dynamic process of transcriptional regulation (Jaskiewicz et al., 2011; Xu et al., 2015; Ramirez-Prado et al., 2018). To test whether S and IR varieties have distinctive histone marks that may explain the differential *SABP2 and YDA* transcriptional activation and subsequent downstream gene expression, we examined major histone modifications. Histone H3 lysine 4 and lysine 27 trimethylations (H3K4me3 and H3K27me3, respectively) are key histone modifications involved in transcriptional regulation. H3K4me3 recruits chromatin remodeling factors that open chromatin to favor transcription, while H3K4me27 shuts down transcription by inactivating gene promoters. Their ratio of H3K4me3/H3K4me27 is known to be an indicator of the positive or negative regulation in gene expression (Jaskiewicz et al., 2011; Ramirez-Prado et al., 2018). In this study, the IR varieties had significantly higher H3K4me3/H3K27me3 ratios than the S variety in noninoculated leaves. After *P. xanthii* inoculation, significant differences were found also in H3K4me3/H3K27me3 ratio in both Otto and Cordelia varieties compared to Kompo (Figure 7, A–C). Examination of the H3K4me3/H3K27me3 ratio in all varieties with and without *P. xanthii* inoculation demonstrated that in noninoculated leaves there was up to 2 times enrichment of HeK4me3 histone marks in the IR varieties compared to S. After *P. xanthii* inoculation, the ratio increased in Kompo plants, which is consistent with chromatin activation for induction of defense mechanisms (Figure 7C). Even though the H3K4me3/H3K27me3 ratio was decreased in Otto variety, the ratio was significantly different compared to Kompo inoculated plants (Figure 7C). Overall, our results show that the IR varieties are endowed with an epigenetic background that modulates transcription suggesting that there is a link between epigenome and the transcription of key resistance-related genes, thus keeping plants in a primed state for defense against pathogen attack.

These findings made the authors speculate that there could be a strong association between *SABP2* and *YDA* Pm-0 genes and the presence of differential histone modifications in each variety. To test this hypothesis, the H3K4me3 and H3K27me3 levels in the gene body of *SABP2* and near the start of the *YDA* coding region were analyzed using chromatin immunoprecipitation followed by RT-qPCR (ChIP-RT-qPCR; Figure 7, D–F). Detection of H3K4me3 levels on the *SABP2* and *YDA* genomic regions revealed significant enrichment in the IR varieties compared to the S variety, suggesting positive transcriptional modulation of these regions (Figure 7, E and F). Detection of H3K27me3 levels showed no significant differences in both regions for all varieties. Collectively, our results demonstrate differences in the enrichment of histone modifications marks on *YDA and SABP2* between S and IR varieties that positively influence gene transcription of these Pm-0 genes. Also, the ChIP results are consistent with our gene expression analyses and the H3K4me3 enrichment detected in the IR varieties (Figure 7A) and clearly demonstrate the transcriptional upregulation and activation of the SA pathway in the IR varieties prior to *P. xanthii* infection.

## Discussion

Obligate biotrophs, such as PM fungi, are a class of plant pathogens that cause infectious diseases, fully depend on their living host for nutrient exchange, and have evolved sophisticated mechanisms to unlock access to plant resources. In response, plants have evolved refined mechanisms to fine tune the activation of specific resistance mechanisms in response to pathogen infection. Cucurbits are the group of plants that are most severely affected by PM worldwide and among the causing agents of PM, *P. xanthii* is prevailing in the Mediterranean basin (Pérez-García et al., 2009; Miazzi et al., 2011). Since the exclusive biotrophic lifestyle of *P. xanthii* limits disease study by fungus molecular manipulation, there is indispensable need to explore the disease from plant’s perspective by understanding the nature of resistance.

Recent advances in deciphering plant–pathogen interactions have made apparent the coordinated action of transcriptional reprogramming in combination with alterations on chromatin marks at defense-associated loci that are often required to selectively activate immune-related genes (Luna et al., 2011). Histone modifications are epigenetic chromatin marks that potentially provide a reversible and dynamic way to regulate many biological processes including plant immunity. In the study reported here, we discovered the differential expression of histone epigenetic marks, that influence gene transcription, in IR plants and the enriched H3K4me3 residue deposition on *SABP2* and *YDA*, Pm-0 genes, positively regulating the transcription of these genes. Additionally, GO term analysis revealed significant GOs that associate transcription with chromatin organization and epigenetic regulation. We suggest that the altered epigenetic background positively modulates defense mechanisms by setting the IR plants in a primed state with induced SAR responses prior to infection by *P. xanthii*.

The significance of the primed defense mechanism was demonstrated by physiological and molecular evidence. First, callose, a typical SAR response when plants are subjected to pathogen attack (Vlot et al., 2009), was evaluated. Callose was deposited in abundance in IR leaves not only after pathogen inoculation but especially in noninoculated IR leaves, suggesting that IR varieties exhibited a pathogen-related response prior to pathogen attack. Assessment of the endogenous SA levels in the noninoculated IR plants confirmed this condition. SA plays a critical role in defense signaling since is required for both basal defense and SAR (Pokotylo et al., 2019). Interestingly, SA levels were not increased in Cordelia after pathogen inoculation, as in the other varieties, even though gene expression of *SABP2*, *PR1*, and *GST* was upregulated. One could speculate that Cordelia, either responds faster to maintain immune homeostasis to better withstand pathogen infection leading to faster assimilation, or other hormone pathways that participate in induced defense are also activated, balancing SA accumulation. Either speculation should be further examined to decipher the underlying mechanisms.

The *SABP2* Pm-0 gene encodes for a gene with MeSA esterase activity. *SABP2* converts inactive MeSA into SA in distal tissues, a conversion that is required for SAR signal perception. Subsequently, SA methyltransferase converts SA to MeSA and another round of SA conversion starts (Vlot et al., 2008). The enriched H3K4me3 residues on *SABP2* genomic region indicate an open accessible chromatin for immediate transcription activation. The observed transcript accumulation of *SABP2* in the noninoculated IR plants agrees with the open chromatin state (Figures 3 and 7).

In addition to callose deposition, we evaluated stomatal and gas exchange parameters revealing substantial differences between S and IR varieties. We observed a profound decrease in stomatal density on the IR plants compared to S. Stomatal area and index on IR plants also highly decreased compared to S in noninoculated plants, while pathogen inoculation decreased the area of Kompo stomata only. Additionally, we did not detect any substantial differences in gas exchange parameters prior and after inoculation in the IR plants, except g*_st_ and E* values in Otto. On the other hand, PM reduced gas exchange parameters in S plants as likewise were observed on grapevine (Moriondo et al., 2005). *YDA* Pm-0 gene is a negative regulator of stomatal development and patterning assisting in plants’ response to abiotic and biotic factors (Sopeña-Torres et al., 2018). The stomatal differences in the IR leaves could be effectively attributed to the observed *YDA* transcript accumulation in the noninoculated IR plants, which is associated with the enriched H3K4me3 residues on its genomic region (Figures 5 and 7). *YDA* is an emerging regulator of plant’s immune responses and in combination with MAPK cascade has been suggested to participate not only in stomatal patterning but also in regulation of immunity and resistance to fungi, among other pathogens in Arabidopsis (Sopeña-Torres et al., 2018), corroborating our results of *YDA and MMK1* increased expression. Moreover, a combine action of SA and MAPK cascades has been proposed, since they can act upstream of each other, with some MAPK cascades triggering SA activity, or SA triggering MAPK cascades (Vlot et al., 2009) demonstrating the multifaceted feature of defense responses.

Examination of marker genes from different hormonal pathways that participate in defense mechanisms, outlined the primed state with induced SAR responses in the IR plants. The enhanced state of the IR plants was also validated by the observed defense-related GO term enrichment. Significant GO terms related to defense responses and the SA pathway were detected in the upregulated genes of the IR varieties. During the onset of SAR responses, a cascade of molecular events is activated. As starting point, one could consider *NPR1*, a pivotal SA receptor and SAR response key regulator, that translocates from cytoplasm to the nucleus to physically interact with transcription factors for downstream induction of defense response gene expression (Backer et al., 2019). *NPR1* and SA marker genes’ expression was significantly increased especially in the noninoculated IR leaves, indicating the primed state with induced SAR responses. Indeed, expression analysis of the PTI and SAR marker gene *WRK29* agreed with the primed state of the IR plants. Furthermore, the increased expression of *WRKY29* is associated with the detected elevated H3K4me3 marks on chromatin of the IR plants (Figure 7A), indicating the tight association between open chromatin state and gene defense priming. Our data agree with evidence showing *WRKY29* serving as a marker for accessible chromatin during SAR (Jaskiewicz et al., 2011).

Another difference between the S and the IR plants was the diverse accumulation of defense compounds. In the noninoculated IR plants, we identified key classes such as flavones, terpenes, and alkaloids. Moreover, the class of HCAs was highly accumulated in both treatments. HCAs include conjugated polyamines, such as *p*-coumaric acid and caffeic acid, which play vital role in plant’s defense responses since they are deposited between primary cell wall and plasmalemma increasing cell wall thickness and reducing the ability of fungal pathogens to penetrate and infect cells (Macoy et al., 2015). Precursor HCAs molecules, such as polyamines, can induce callose deposition and defense responses in Arabidopsis (Liu et al., 2019). Interestingly, HCAs derive partly from the phenylalanine pathway like SA, and has been shown that HCAs accumulation is accompanied by SA increase and induced resistance in transgenic tomato after *P.* *syringae* pv. *tomato* infection (Campos et al., 2014). Notably, we detected substantial accumulation of the antimicrobial metabolites coumarins in noninoculated IR plants, which are shown to be connected with the SA defense pathway also (Pastı’rová et al., 2004). Plants utilize an integration of defense mechanisms to resist against pathogens.

The examination of *SAPB2 and YDA* Pm-0 genes has set the basis for understanding how the recently emerged Pm-0 resistance locus against PM functions. Our results showed that these genes modulate primed state with induced SAR responses of courgette plants with activated defense to encounter imminent *P. xanthii* attack and brought insights on deciphering plant’s responses against this highly notorious biotrophic pathogen. The resistance mechanism is a result of a combined Pm-0 genes’ action and is highly significant to explore further the underlying mechanisms to understand defense responses and set new targets of resistance.

## Materials and methods

### Plant material and growth conditions

Three courgette (*Cucurbita pepo* L. var. *cylindrica*) varieties with different levels of genetic resistance to PM were used; the IR *C. pepo* F1 hybrids Otto and Cordelia (Syngenta AG, Switzerland) and the Susceptible (S) cultivar (cv) Kompokolokitho (Kompo, No. 951, Hellenic Agricultural Organization-Demeter, Institute of viticulture and vegetable crops, Pirgos, Greece). Seeds of all varieties were placed in individual 15-cm pots containing sterile peat substrate for germination, and 1 week later they were transplanted to 2-L pots. When plants developed three to four true leaves, they were transplanted into 20-L pots until the end of the experiments. All plants were grown in controlled greenhouse conditions (16-h light/8-h dark, 60% relative humidity, 22°C) under a completely randomized, split-plot design with six blocks/replicates (24 plants).

### Pathogenicity assays

*Podosphaera* *xanthii* inoculations were carried out on 21-d-old soil-grown plants by spraying the plants with a suspension of 1.5 × 10^5^ conidia mL^−1^ of the fungus. Disease severity in inoculated plants was estimated by visual assessment of percent infected leaf area, following the principles of EPPO STANDARD PP1-057-3 “Powdery mildew of cucurbits and other vegetables” (EPPO, 2005).

### Marker-assisted genotyping

Validation of each genetic background was performed according to Holdsworth et al. (2016). Briefly, genomic DNA from each variety was extracted from 50-mg leaf tissue using the CTAB method. About 20 ng of DNA template was used for amplification of the NBS_S9_1495924 and S9_1539675 CAPS markers. The markers were amplified on a MiniAmp Plus Thermal Cycler (Applied Biosystems, Waltham, MA, USA). The PCR products were subsequently digested with the restriction enzymes HaeIII and MspI under the same digestion conditions. The full digests were analyzed in 1.5% (w/v) agarose gel stained with Midori Green Advance (Nippon Genetics, Tokyo, Japan).

### Leaf gas exchange parameters

Net *A*, *gs*, *E*, and *C*i were measured 3 times per plant per treatment, using both the third and fourth fully developed leaves from the shoot apex. The measurements were carried out with Li-6400XT (Li-COR) portable photosynthesis measuring system under steady light intensity (800 μmol m^−2^s^−1^) and CO_2_ concentration (400 mg L^−1^), while leaf temperature ranged between 27.5° C and 29.8°C.

### Confocal laser scanning microscopy

Callose deposition was assessed in leaf samples that were mounted between a microscope slide and coverslip in water. Z series were captured using TCS SP8 confocal laser-scanning microscope (Leica). Callose was stained with Aniline blue (Sigma Aldrich) and was excited at 405 nm by a diode laser. Emission filtering was achieved using a 472- to 550-nm bandpass filter for Aniline blue. Stomata were stained with Safranin-O (Sigma Aldrich, St Louis, MO, USA) which was excited with the 520 nm line of TCS SP8 White Light laser (WLL). Emission filtering was achieved using a 570- to 600-nm bandpass filter. *Podosphaera* *xanthii* conidia were visualized with a chitin-binding lectin conjugated with a green fluorescent dye, WGA-488 (Biotium Fremont, CA, USA). WGA-488 was excited with the 488 nm line of WLL and emission filtering was achieved using a 510- to 540-nm bandpass filter. During image acquisition each line was scanned 5 times and averaged. Image processing, including shadow 3D projection, in silico cross-section, maximum intensity 3D reconstruction, and surface rendering, was performed using integral functions of the LAS X (Leica, Wetzlar, Germany) operating software.

### RNAseq data analysis

The reads were mapped to the *C.* *pepo* subsp pepo reference genome version 4.1 (Cpepo_v4.1, http://cucurbitgenomics.org/) using STAR version 2.5.3.a (Dobin et al., 2013). Expression counts were generated using the R function summarizeOverlaps from the package HTSeq in union mode on exons. Differential expression analyses were performed using the R package DESeq2 version 1.32.0 (Love et al., 2014). Genes with absolute log2FoldChange ±1 were considered as differentially expressed. GO enrichment analysis of DEGs was performed using the R package topGO (Alexa and Rahnenfuhrer, 2016). Fold enrichment is obtained through comparing the background frequency of total genes annotated to that term to the sample frequency representing the number of genes inputted that fall under the same term.

### RT-qPCR

Artificial inoculation with *P. xanthii* conidia was performed as described above. Leaf material from the controlled greenhouse experiment varieties was collected 36 hpi. The second and third youngest fully expanded leaves were collected and pooled for each one of three individual plants per treatment, considering each plant as one biological sample. Plant material was frozen in liquid nitrogen and stored at −80°C until use.

Total RNA was extracted using NucleoZOL (Macherey-Nagel) and cDNA was synthesized using 1 μg of total RNA and the PrimeScript RT reagent Kit with gDNA Eraser (Takara Bio, Shiga, Japan) according to the manufacturer’s instructions. Real-time PCR was performed using StepOnePlus thermal cycler real-time system (Applied Biosystems) and KAPA SYBR FAST qPCR kit master mix (KAPA Biosystems, Woburn, MA, USA) containing 200 nM of each primer and 1 μL cDNA template (1/20th of cDNA reaction volume). The following conditions were applied: 10 s at 95°C, followed by 40 cycles of 95°C for 5 s and 60°C for 30 s, with a final 5 s at 95°C and 30 s at 60°C. The reactions were run in duplicate and repeated 3 times. The followed thermal amplification and melt curve profile, were as indicated by the manufacturers. Nonreverse transcribed samples and nontemplate controls were also included in the relevant PCR runs. The relative gene expression was determined with the ΔΔ_CT_ method. The information of primers used in this study is provided in Supplemental Table S4.

### Western blot analysis

Proteins were extracted from leaves of courgette varieties that were water-treated and or inoculated with *P. xanthii* conidia and collected 36 hpi. The tissue was grounded in liquid nitrogen, and total proteins were isolated with an NP-40, SDS, Triton X-100 extraction buffer, and 2 min total sonication steps. Equal amounts of proteins were resolved on a 12% SDS–polyacrylamide gel and transferred to Immobilon-P membrane (Millipore, Darmstadt, Germany). The primary antibodies used for immunoblotting were rabbit anti-H3K4me3 (Cell Signaling, Danvers, MA, USA), rabbit anti-H3K27me3 (Cell Signaling), and rabbit anti-H3 (Agrisera, Vännäs, Sweden). The membranes were washed and incubated with HRP-conjugated goat anti-rabbit IgG (Carl Roth). The chemiluminescence signals were detected with Clarity Western ECL Substrate (Bio-Rad, Hercules, CA, USA), and imaging was performed with ChemiDoc imaging system (Bio-Rad). Band intensity of the chemiluminescence signal from three biological and two technical western blots for each sample in each western blot was analyzed and averaged with Image Lab Software (Biorad).

### ChIP

ChIP was performed as described previously by Bowler et al. (2004). Briefly, two 21-d-old courgette leaves from each variety were freshly collected and fixed. Chromatin was isolated from 1 g of tissue. Immunoprecipitation was performed with either anti-H3K4me3, or anti-H3K27me3 (Millipore) antibody. Fold enrichment of immunoprecipitated chromatin for each target gene was plotted according to the ΔΔ_CT_ method by RT-qPCR. The CT value for the antibody sample (+AB) and for no antibody control (−AB) was independently subtracted from the CT value of the corresponding input to find Δ_CT_. Then Δ_CT_^–AB^ was subtracted from Δ_CT_^+AB^ to get the ΔΔ_CT_ for each sample and 2^−^^ΔΔCT^ was plotted (Mukhopadhyay et al., 2008). The selectivity of the immunoprecipitated DNA was tested with the euchromatic *EFL1a* gene (Supplemental Figure S1). Primers used in this study are mentioned in Supplemental Table S4.

### Metabolite analysis

For HR-MS Metabolomics analysis, three 21-d-old water-treated (noninoculated, W) plants or inoculated with *P. xanthii* from all varieties were collected ground in liquid nitrogen, and freeze-dried. The extracts were injected in a Q-Exactive Orbitrap High-Resolution Mass Spectrometry (HR–MS) Platform (Thermo Scientific, Waltham, MA, USA) following a metabolomics protocol as previously described (Nikolaivits et al., 2019). The MS data were subjected to multivariate statistical analysis using SIMCA P + 11.5 software (Umetrics, Umea). The variables that exhibited variable importance in projection scoring ˃1 were verified by *t* test using a *P*-value at ≤0.05. These variables were forwarded to annotation using online databases such as Chemspider, Metlin, Lipid Maps, and so on applying *m/z* tolerance of 5 ppm and taking into consideration the isotopic and MS/MS fragmentation pattern.

The samples of the HR–MS Metabolomics analysis were used for quantification of SA levels as previously described (Margaritopoulou et al., 2020).

### Statistical analysis

Statistical significances based on one-way and two-way analysis of variance (ANOVA)(Dunnett’s and Tukey’s posthoc tests, respectively) were performed with Prism (GraphPad). All the *P*-values of the two-way ANOVA analyses are presented in Supplemental Table S5.

## Supplemental data

The following materials are available in the online version of this article.

**Supplemental Figure S1.** Courgette disease infection and *P. xanthii* conidia germination.

**Supplemental Figure S2.** *Podosphaera xanthii* growth on courgette leaves as observed with optical and confocal microscopy.

**Supplemental Figure S3.** Metabolite analyses profiles.

**Supplemental Figure S4.** Aniline blue stained callose depositions.

**Supplemental Figure S5.** Transcriptomic analyses profiles.

**Supplemental Figure S6.** Gas exchange parameters.

**Supplemental Figure S7.** qPCR for the euchromatic EFL1a gene in ChIP input samples and final chromatin samples after immunoprecipitation in ChIP assay.

**Supplemental Table S1.** Annotations of the detected metabolites in the water treated plants.

**Supplemental Table S2.** Annotations of the detected metabolites in plants after pathogen inoculation.

**Supplemental Table S3.** Pm-0-interval putative genes in courgette.

**Supplemental Table S4.** Primers used in this study.

**Supplemental Table S5.** *P*-values of the two-way ANOVA analyses.

**Supplemental Data Set S1.** RNA-seq data for Cordelia to Kompo variety comparison.

**Supplemental Data Set S2.** RNA-seq data for Otto to Kompo variety comparison.

**Supplemental Data Set S3.** RNA-seq data for Cordelia to otto variety comparison.

**Supplemental Data Set S4.** Counts of technical and biological replicate samples analyzed by RNA-seq.

**Supplemental Data Set S5.** GO-term analyses of the upregulated DEGs.

**Supplemental Data Set S6.** GO-term analyses of the downregulated DEGs.

## Supplementary Material

kiab453_Supplementary_Data

## Notes

T.M., D.K., and E.M. designed the project, coordinated data analysis, and manuscript preparation; T.M. performed molecular, microscopic assays, and transcriptomic analyses; E.M. and A.E.V. performed pathogenic response assays; D.Ko. performed molecular and physiological analyses; I.E.P. performed gas exchange measurements; C.A. performed SA analyses; E.M. and A.T. performed metabolomic assays; T.M. wrote the original draft of the manuscript; T.M., D.K., C.L., and E.M. critically reviewed the manuscript, all authors approved the final version of the manuscript for publication.

The author responsible for distribution of materials integral to the findings presented in this article in accordance with the policy described in the Instructions for Authors (https://academic.oup.com/plphys/pages/general-instructions) is Theoni Margaritopoulou (th.margaritopoulou@bpi.gr).
