# Corrigendum to: Enriched HeK4me3 marks at Pm-0 resistance-related genes prime courgette against *Podosphaera xanthii*

**DOI:** 10.1093/plphys/kiab502

**Published:** 2021-11-11

**Authors:** Theoni Margaritopoulou, Dimosthenis Kizis, Dimitris Kotopoulis, Ioannis E Papadakis, Christos Anagnostopoulos, Eirini Baira, Aikaterini Termentzi, Aikaterini-Eleni Vichou, Carlo Leifert, Emilia Markellou

*Plant Physiology*, kiab453, https://doi.org/10.1093/plphys/kiab453

The paper above contained the following incorrect reference:

**Samakovli D, Tichá T, Vavrdová T, Ovečka M, Luptovčiak I, Zapletalová V, Kuchařová A, Křenek P, Krasylenko Y, Margaritopoulou T** (2020) YODA-HSP90 module regulates phosphorylation-dependent inactivation of SP EECHLESS to control stomatal development under acute heat stress in Arabidopsis. Mol Plant Pathol **13**: 612–633

The corrected reference is as follows:
