# Correction to: Enriched H3K4me3 marks at Pm-0 resistance-related genes prime courgette against *Podosphaera xanthii*

**DOI:** 10.1093/plphys/kiac446

**Published:** 2022-10-06

This is a correction to: Theoni Margaritopoulou, Dimosthenis Kizis, Dimitris Kotopoulis, Ioannis E Papadakis, Christos Anagnostopoulos, Eirini Baira, Aikaterini Termentzi, Aikaterini-Eleni Vichou, Carlo Leifert, Emilia Markellou, Enriched H3K4me3 marks at Pm-0 resistance-related genes prime courgette against *Podosphaera xanthii*, Plant Physiology, Volume 188, Issue 1, January 2022, Pages 576–592, https://doi.org/10.1093/plphys/kiab453

In the originally published version of this manuscript, there was an error in the title, whereby ‘H3K4me3’ was given as ‘HeK4me3’.

This error has been corrected.

