# Supplementary material for: Enriched H3K4me3 marks at Pm-0 resistance-related genes prime courgette against Podosphaera xanthii
Source: Plant Physiol. 2021 Sep 21;188(1):576–92. doi: 10.1093/plphys/kiab453 (PMC8774738; doi:10.1093/plphys/kiab453)
Supplement: kiab453_Supplementary_Data [file kiab453_supplementary_data.zip › kiab453-suppl_data/Supplemental_Data.pdf]

Supplemental figure Legends

Supplemental Figure S1. Courgette disease infection and *P.xanthii* conidia germination. A and B, 4 w-old courgette leaves (A) and 5 w-old plants (B) from each variety demonstrating PM disease progress. Whole 2-wk-old courgette plants of each variety were artificially inoculated with an aqueous conidial suspension of *P. xanthii*, at a titre of 1.5 x 10^5 CFU ml^{-1}. Leaves were photographed at 7 dpi and total plants were photographed 14 dpi. C, variety effect on conidia germination. Germination was evaluated at 12 hpi on courgette leaves. At least three leaf areas from three different leaves of each variety were stained with Lactophenol blue and the germinated conidia were counted under the microscope. Data represented the mean of four measurements of 125 conidia ± SEM (n=4). Asterisks represent statistically significant differences (***P<0.001) as determined by one-way ANOVA followed by Dunnett's post hoc test.

Supplemental Figure S2. *P. xanthii* growth on courgette leaves as observed with optical and confocal microscopy.

A, Lactophenol blue staining of germinated conidia on Kompo leaves at different developmental stages 1. Germinated appressorium (app), 2. Developed first primary hyphae (ph1), 3. Developed second primary hyphae (ph2), 4. Developed third primary hyphae (ph3). co: conidium. B, Representative *P. xanthii* germinated conidia on Kompo leaf 36 hpi with three developed primary hyphae. Conidia on Kompo leaf were stained with WGA-488 for visualisation. Right micrograph shows a z-stack of optical sections for visualisation of haustorium (ha) that has penetrated inside the cell. Colour scale bar shows the depth size of the z-stack in μm. Approximately at 15μm is the top of the epidermal cells. Abbreviations are as in (A). C, Representative *P. xanthii* germinated conidia on Kompo, Otto and Cordelia leaves 36 hpi. Micrographs show z-stacks of optical sections for visualisation of haustorium that has penetrated inside the cell. Conidia on Kompo leaf were stained with WGA-488 and leaves were stained with Safranin-O for visualization of cell walls. A and B are the same micrographs with the addition in B of depth coding presentation. Colour scale bar shows the depth size of the z-stack in μm. Approximately at 15μm is the top of the epidermal cells.

Supplemental Figure S3. Profiles of the metabolite analyses

Score plots from PCA, PLS-DA, permutation test and S-plots after the processing procedure of tissues samples from IR plants (Cordelia/Otto, red triangles) and S plants (Kompo, black

triangles) employing UHPLC–HRMS (Orbitrap) analysis, in both negative and positive ion mode. A, Mass spectrometric analysis in the negative ion mode for the correlation of water-treated IR plants to S plants. The analysis in the positive ion mode did not show any variation. B and C, Mass spectrometric analysis in the negative (B), and positive (C) ion mode for the correlation of *P. xanthii* inoculated IR plants to S plants. Permutation testing allowing of 100 permutations of the PLS-DA model showed that the goodness of fit and predictive ability (R2/Q2) of the original model was higher than those of the permuted models. S-plot of PLS-DA showed the relative contribution of each variable to clustering between the IR and the S group. ESI: Electrospray ionization, PCA: Principal Component Analysis, PLS-DA: Partial Least Squares–Discriminant Analysis.

Supplemental Figure S4. Aniline blue stained callose depositions in Kompo, Otto and Cordelia leaves before (W samples) and 36 hpi with *P. xanthii* conidia suspension (WX). Examples of circular-like granules of callose deposits are marked with arrows. Examination of callose deposits on leaves was performed by tile scanning with Navigator module of Leica LASX software. The scan dimensions are depicted on the left down side of each picture. At least 3 different areas of 3 leaves from each sample were scanned.

Supplemental Figure S5. Profiles of the varieties' transcriptomes

A, Principal component analyses of differentially expressed genes identified in all samples. B, Validation of RNAseq results by comparing the up- and downregulated genes of the expression analyses performed in Figure 3 to the log2FC values of the transcriptome analysis.

Supplemental Figure S6. Gas exchange parameters.

A, Water use efficiency (WUE) was computed as the ratio between *A* and *E* of each one measurement presented in Figure 6. Similarly, B, intrinsic WUE (iWUE) was calculated by dividing the *A* with g_{st} values of each one recorded measurement, respectively. Data, as in Figure 6, represent the mean of four measurements from 3 biological replicates ± SEM (n=3). Significant differences for WUE were obtained between varieties (*P*=0.0050), *P. xanthii* inoculation (*P*=0.0194) and the interaction of variety x inoculation (*P*=0.0473). Significant differences for iWUE were obtained between varieties (P=0.0030), *P. xanthii* inoculation (*P*=0.0003) and the interaction of variety x inoculation (*P*=0.0031). Means of different varieties that received the same inoculation treatments, indicated with lower case letters and *P. xanthii*

inoculated and non-inoculated plants of the same variety indicated with different capital letter, are significantly different according to Tukey's multiple comparisons test ($P<0.05$).

Supplemental Figure S7. qPCR for the euchromatic *EFL1a* gene in ChIP input samples and final chromatin samples after immunoprecipitation in ChIP assay.

EFL1a DNA is highly detected in the isolated chromatin sample from each variety (Kompo, Otto, Cordelia input samples), while no amplicon is detected in the chromatin samples after the immunoprecipitation with no antibody (m_ChIP samples) or H3K4me3 (4_ChIP samples) and H3K27me3 antibodies (27_ChIP samples). K: Kompo, O: Otto, Co: Cordelia. The experiment was performed in all three biological replicates of the ChIP assay with similar results.

Supplemental Figure S1

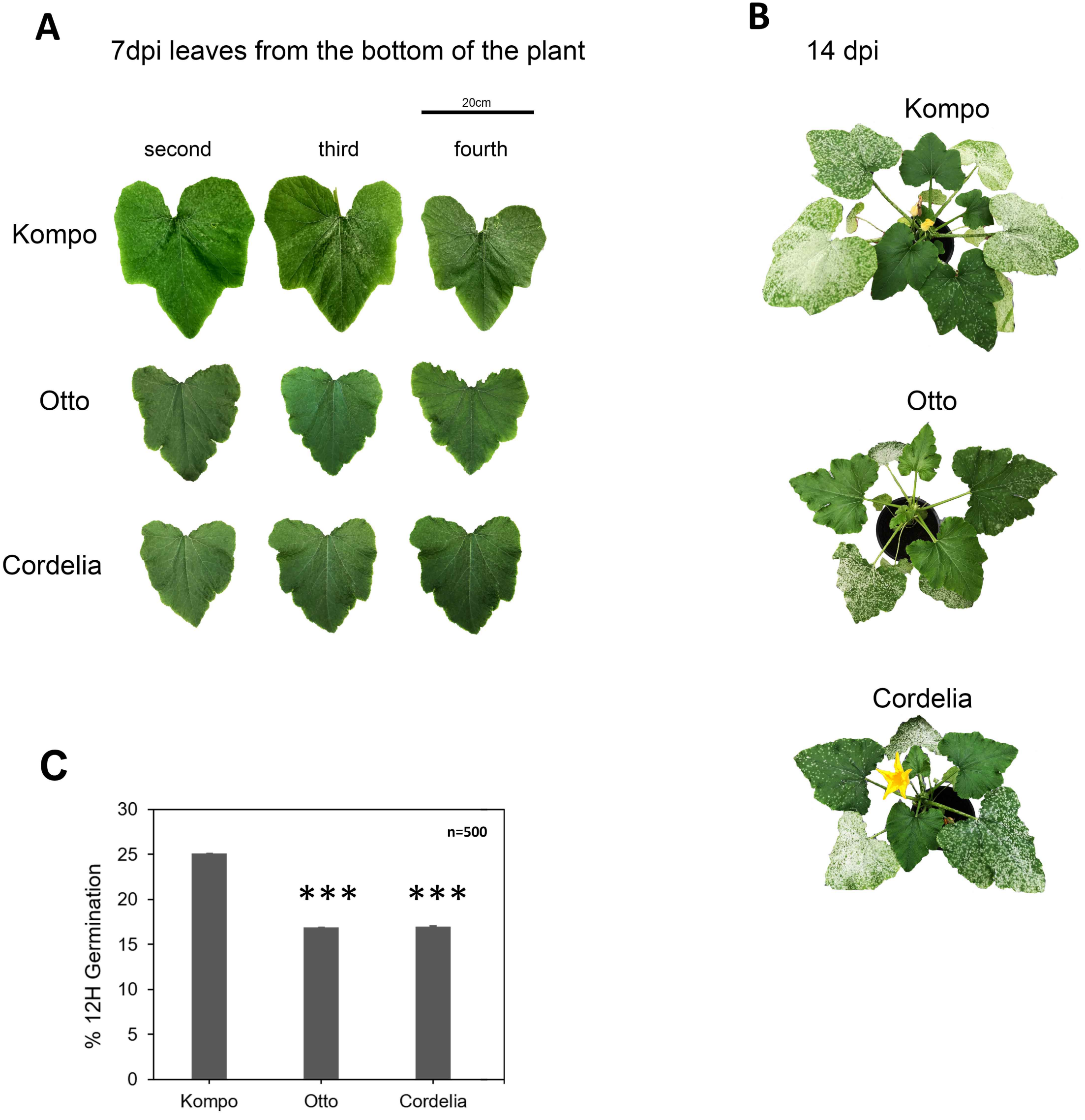

Supplemental Figure S2

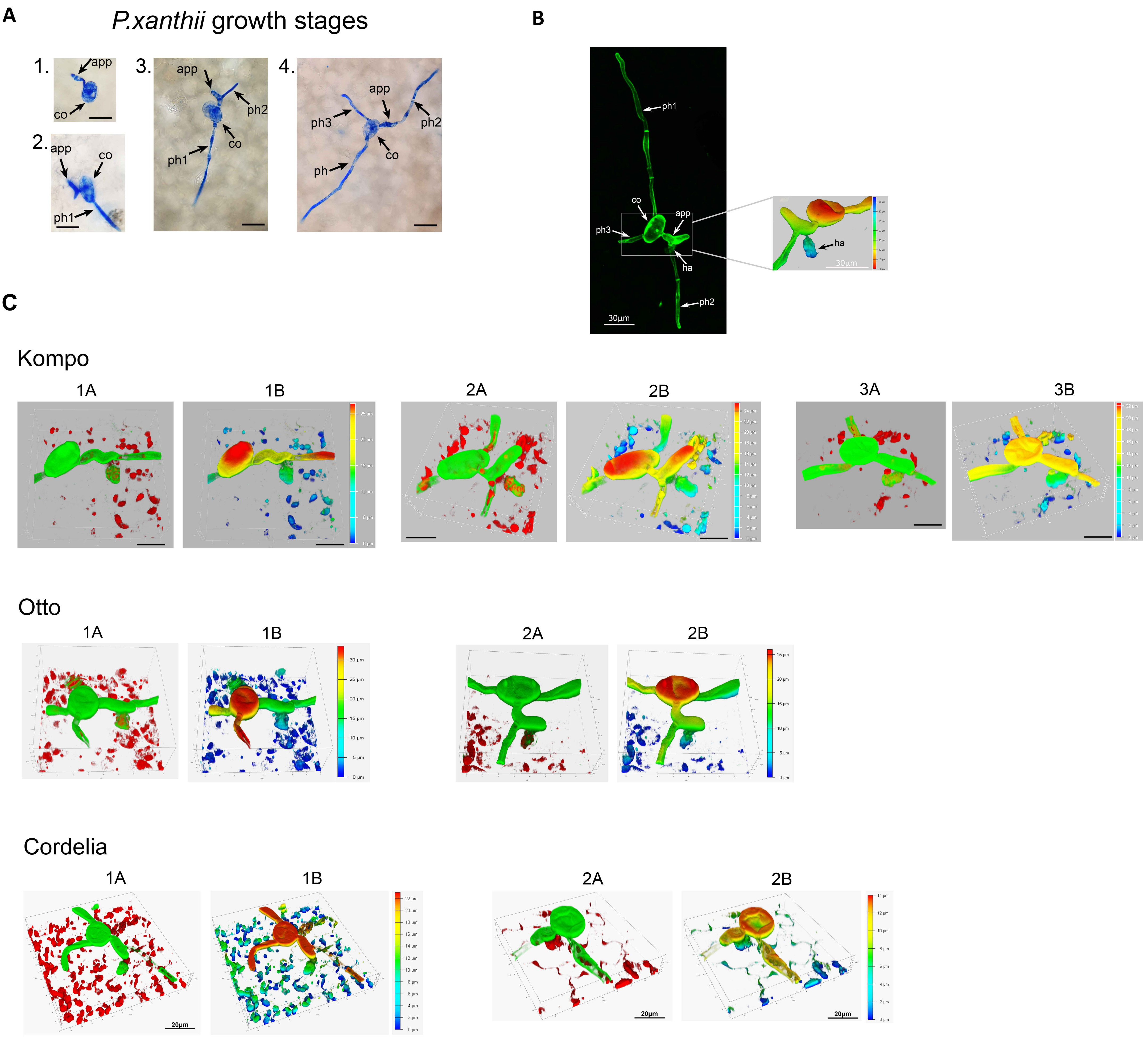

Supplemental Figure S3

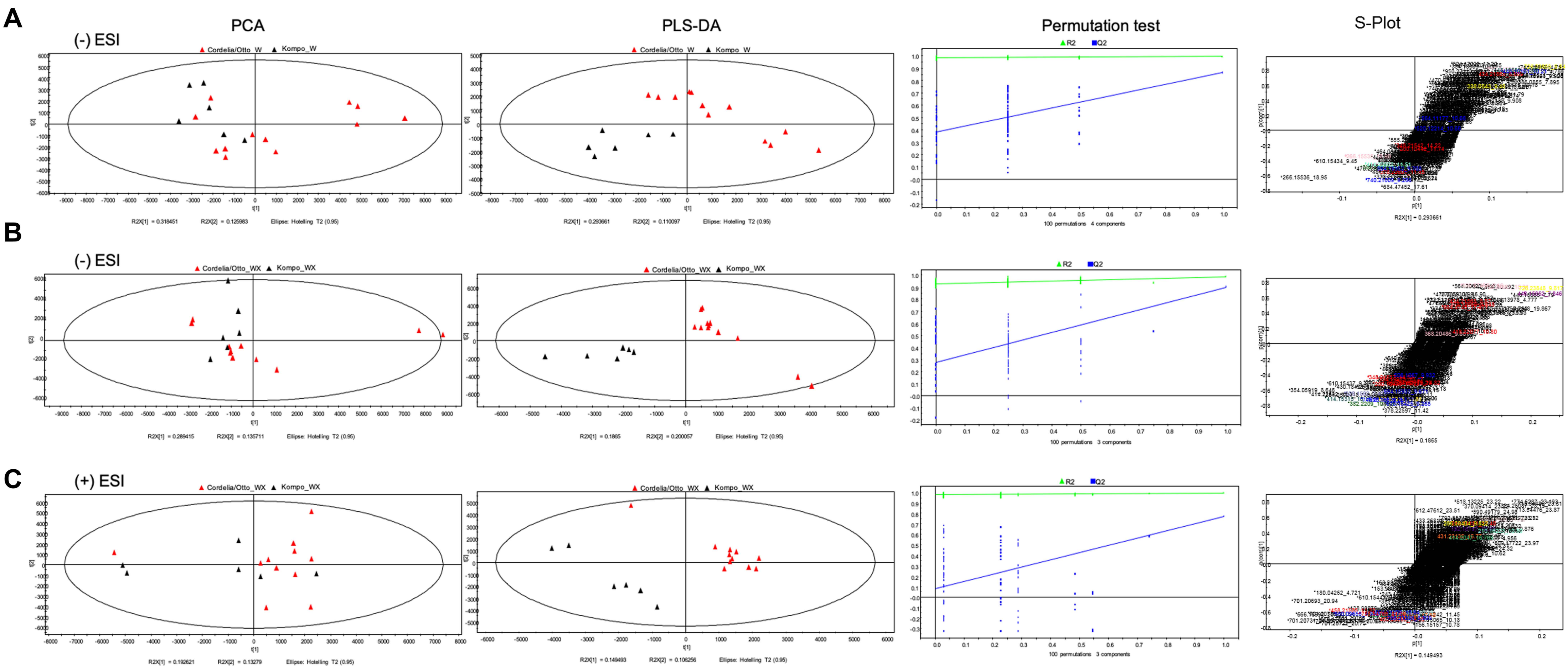

Supplemental Figure S4

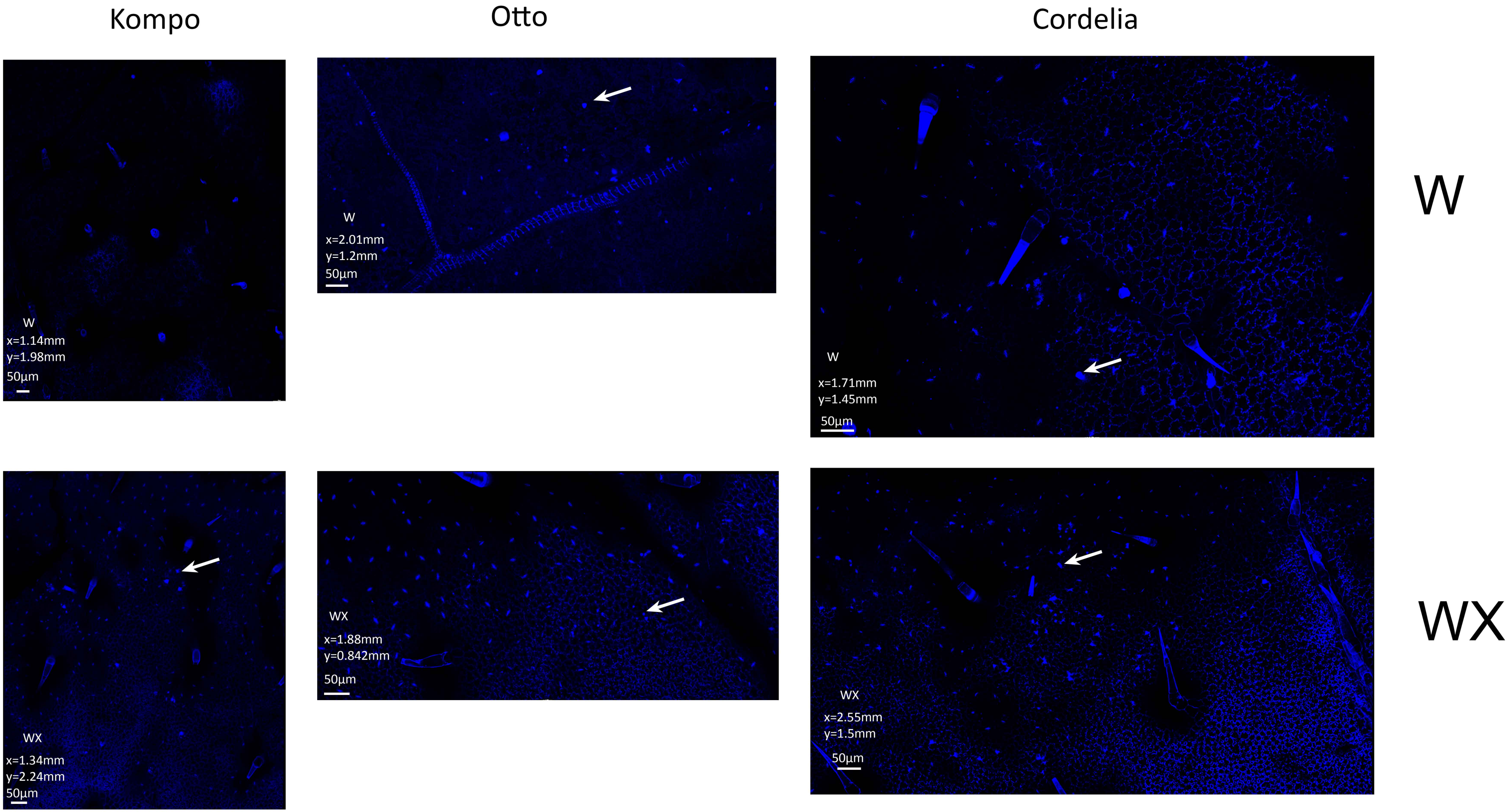

Supplemental Figure S5

A

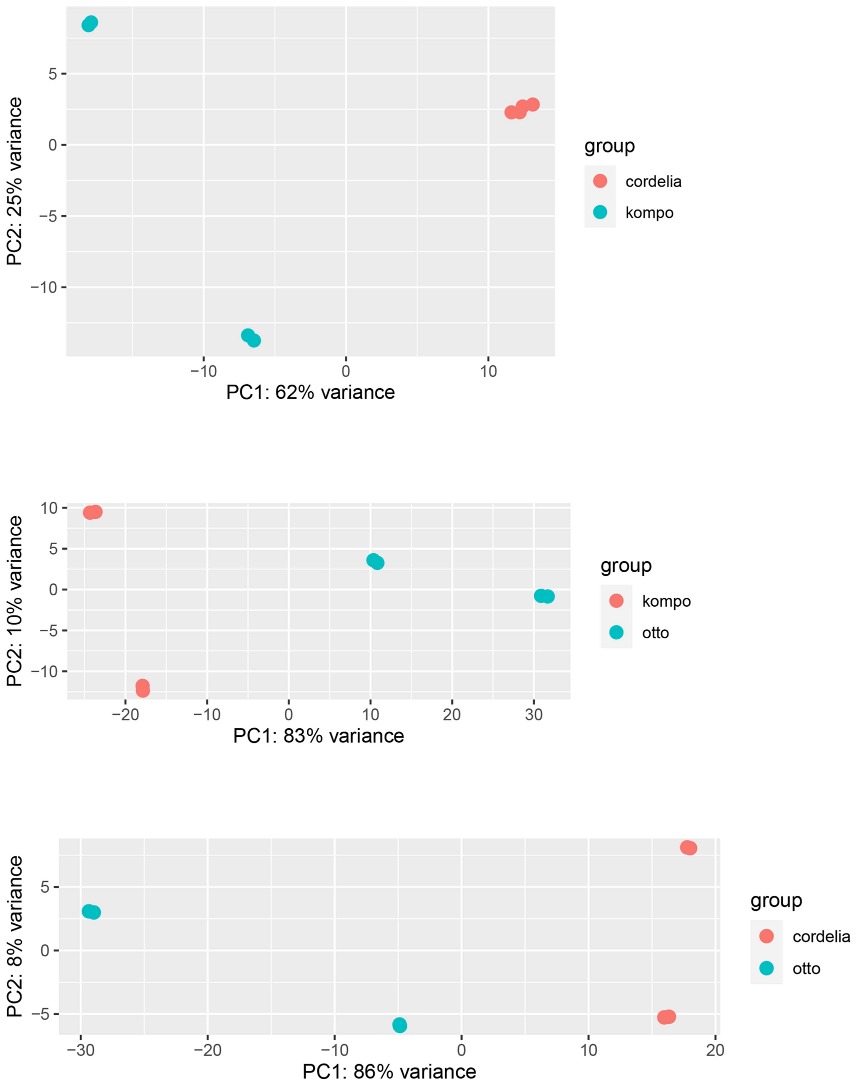

B

log2FC in RNAseq		Cordelia_Kompo		Otto_Kompo	
		LFC	padj	LFC	padj
	Cp4.1LG19g11360.1 (SABP2)	4.879279957	0.00424254	4.14411587	0.009869886
	Cp4.1LG01g00250.1 (WRKY29)			4.142591982	0.007442592
	Cp4.1LG09g06950.1 (NPR1)			0.538647348	0.005739707
	Cp4.1LG13g00640.1 (VSP2)	0.586341236	0.00778607	0.619165197	0.001933589
	Cp4.1LG05g00100.1 (MYC2)	-0.144162979	0.176701349	-0.506255971	0.003665478
	Cp4.1LG15g04130.1 (PR1)	3.705357108	0.00000883	3.111747086	0.000185512
	Cp4.1LG08g03030.1 (GST)			2.026784974	0.002241412
		Cordelia_Kompo		Otto_Kompo	
		LFC		LFC	
log2FC in qPCR relative to EF1a	Cp4.1LG19g11360.1 (SABP2)	1.639092827		2.053038968	
	Cp4.1LG01g00250.1 (WRKY29)			1.881384014	
	Cp4.1LG09g06950.1 (NPR1)			0.951841867	
	Cp4.1LG13g00640.1 (VSP2)	0.799087306		0.763836459	
	Cp4.1LG05g00100.1 (MYC2)	0.471510394		-1.375391229	
	Cp4.1LG15g04130.1 (PR1)	4.958604541		8.08730601	
	Cp4.1LG08g03030.1 (GST)			1.533442496	

Supplemental Figure S6

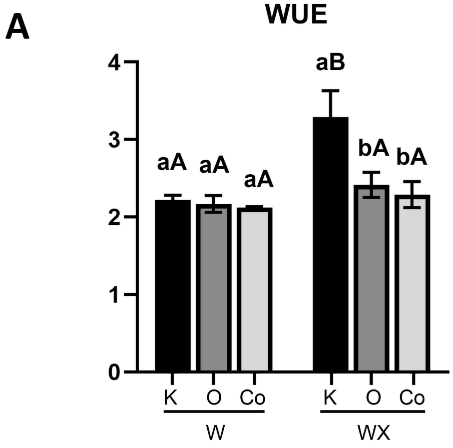

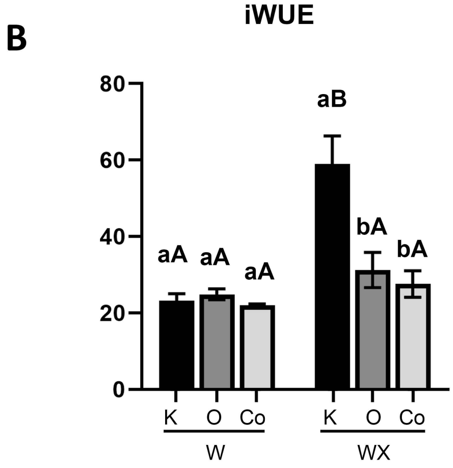

Supplemental Figure S7

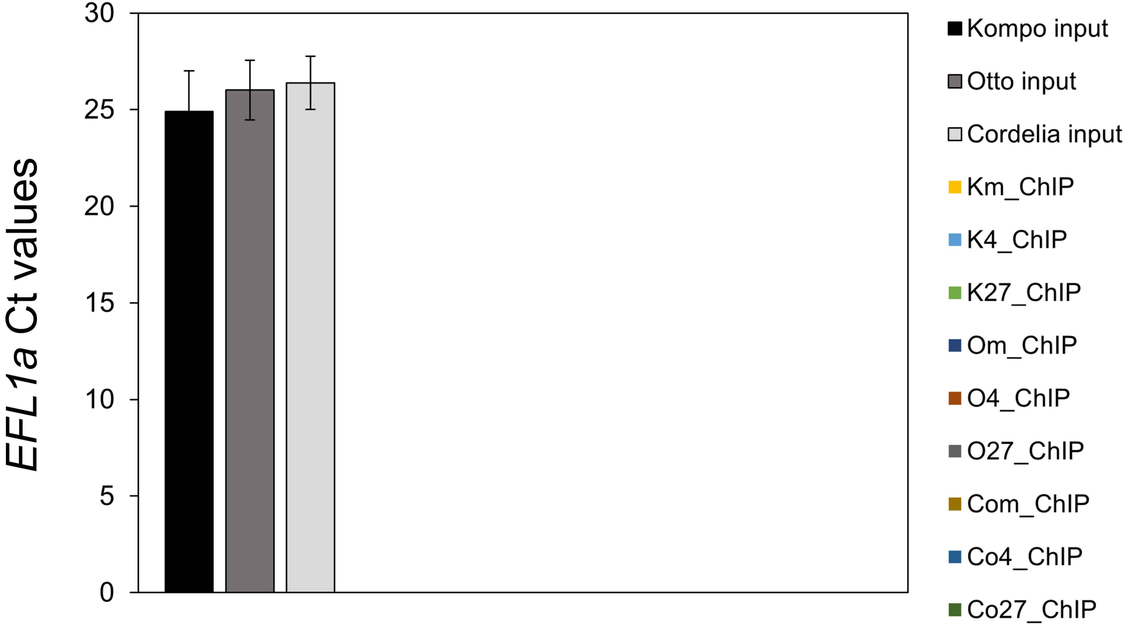

Supplemental Table S1: Annotations of the detected metabolites in the IR (Otto, Cordelia) versus S (Kompo) comparison of water treated (W) control samples

Class	Possible Annotation	Ratio (IR/S)	Vip Scoring	P-value
Fatty acids	Decenedioic acid	0.595592615	1.38528	0.002339107
Fatty acids	2-(2-Butoxyethoxy)ethyl 2-methoxyethyl hexanedioate	0.459981601	2.24746	0.025970687
Fatty acids	Bis(2-{2-[(oxiran-2-yl)methoxy]ethoxy}ethyl) hexanedioate OR 3,6,9,12,15,18-Hexaoxa 1,20-diyl diacrylate	4.016064257	1.22481	0.000751158
Fatty acids	2-(2-Butoxyethoxy)ethyl 2-(2-ethoxyethoxy)ethyl butanedioate	0.320102433	1.44387	0.00040164
Flavones/Flavonols	Isorhamnetin 3-(6''-acetylglucoside) OR Luteolin 7-(2''-glucosyllactate)	0.239406272	1.14799	0.000383894
Flavones/Flavonols	Cyanidin 3-(6''-malylglucoside) OR Petunidin 3-(6''-malonylglucoside) OR Isorhamneti malonylglucoside) OR Larycitrin 3-(4''-malonylrhamnoside)	0.447828034	1.19993	0.004814751
Flavones/Flavonols	Kaempferol 3-rhamnoside-7-glucoside: Kaempferol 7-neohesperidoside OR Kaempfer rhamnoside-7-glucoside	0.211371803	1.06817	0.0095069
Flavones/Flavonols	Astragalin	3.610108303	2.16389	0.002416104
Flavones/Flavonols	Robinin	0.114652603	1.02855	0.034600375
Flavanols	Epigallocatechin/catechin	1.85528757	1.39969	0.001765237
Flavanols	Naringin 6''-rhamnoside	24.3902439	1.15161	0.002059129
Phenolic glycosides	4-Methoxyphenyl 4-O-(b-D-galactopyranosyl)-b-D-glucopyranoside	5.747126437	1.33966	0.003621222
Hydroxycinnnamic acid derivatives	Coumaric acid ester	1.865671642	1.44981	0.006105136
Hydroxycinnnamic acid derivatives	2-O-Caffeoylglucarate	1.862197393	1.82878	1.83525E-08
Hydroxycinnamic acid derivatives	Pulverulentoside I; (1aS,1bS,2S,5aR,6S,6aS)-6-({3-O-Acetyl-6-deoxy-2-O-[(2E)-3-(4-methoxyphenyl)-2-propenoyl]-?-L-mannopyranosyl}oxy)-1a-(hydroxymethyl)-1a,1b,2,5a hexahydrooxireno[4,5]cyclopenta[1,2-c]pyran-2-y l ?-D-glucopyranoside	35.71428571	1.82664	3.89806E-05
Caffeic acid derivatives		0.321233537	2.41464	0.034726267
Fatty aldehydes		0.371057514	2.7658	0.003573172

Supplemental Table S2. Annotations of the detected metabolites in plants after pathogen inoculation

Class	Possible Annotation	Ratio (IR/S)	Vip Scoring	P-value
Fatty acids	Decenedioic acid	0.454959054	2.1624	0.000921896
Fatty acids	5,8,11-Trioxapentadeca-1,15-diyl diacetate	0.667111408	1.19809	0.030228028
Fatty acids	2-(2-Butoxyethoxy)ethyl 2-methoxyethyl hexanedioate	0.659195781	1.5116	0.028746296
Fatty acids	1-O-dodecanoyl-D-glucopyranoside	0.64808814	1.20855	0.046463893
Fatty acids	2,5,8,10,13-Pentaoxaheptadecanoic acid, 9-oxo-, 2-butoxyethyl ester OR 4,8,12,16,20-Pentaoxatricosa-1,22-diene-6,10,14,18-tetrol	0.51203277	1.04047	0.014630128
Fatty acids	Glycerophospho-N-palmitoyl ethanolamine	0.552791598	1.54816	0.000302039
Fatty acids	Diethenyl 6,17-dioxo-7,10,13,16-tetraoxadocosane-1,22-dioate	0.025234683	2.69538	0.026770106
Fatty acids	PC(17:2(9Z,12Z)/17:1(9Z))etc	0.236574403	1.70273	0.008261892
Fatty acids	PA(18:2(9Z,12Z)/17:1(9Z)) etc.	1.865671642	1.21681	0.004403907
Fatty acids	4,8,11,14,17,20,24-Heptaoxaheptacosa-1,26-diyne-6,22-diol	1.953125	2.93482	0.000401514
Fatty acids	Butyl 2-methyl-4,12-dioxo-3,5,8,11,13-pentaoxaheptadecan-1-oate	1.879699248	1.09937	0.000149104
Fatty acids	Bis(2-{2-[(oxiran-2-yl)methoxy]ethoxy}ethyl) hexanedioate OR 3,6,9,12,15,18-Hexaoxaicosane-1,20-diyl diacrylate	4.255319149	1.63117	0.003670733
Fatty acids	12-oxo-10E-dodecenoic acid	1.510574018	1.13653	0.018351691
Flavones/Flavonols	Quercetin 3-O-(6''-acetyl-glucoside) OR Myricetin 3-(2''-acetylrhamnoside)	0.330687831	1.19125	0.001505721
Flavones/Flavonols	Kaempferol 3-rhamnoside-7-glucoside: Kaempferol 7-neohesperidoside OR Kaempferol 3-rhamnoside-7-glucoside	0.15805279	1.13118	0.006964992
Flavones/Flavonols	Rutin	0.211104074	2.51489	0.002975248
Flavones/Flavonols	Quercetin 3-(6''-malonylneohesperidoside)	0.314564328	1.54404	0.000906326
Flavones/Flavonols	Robinin	0.103124678	1.89248	0.021073294
Flavones/Flavonols	Quercetin 3-(6''-malonylglucoside)	0.2	3.29366	0.001870431
Flavones/Flavonols	Cyanidin 3-(6''-malylglucoside) OR Petunidin 3-(6''-malonylglucoside) OR Isorhamnetin 3-(6''-malonylglucoside) OR Larycitrin 3-(4''-malonylrhamnoside)	0.240905806	1.59211	0.005082705

Flavones/Favonols	Cyanidin 3-(2G-glucosylrutinoside) OR Pelargonidin 3-sophoroside-7-glucoside OR Cyanidin 3-sophoroside-5-glucoside OR Pelargonidin 3-gentiotrioside	0.137343771	1.04906	0.000256625
Flavanols	Naringin 6''-rhamnoside	2.824858757	2.41647	0.010110785
Phenolic glycosides	Ptelatoside A	0.337952011	3.65856	0.003448572
Phenolic glycosides	Aloesol 7-glucoside	0.299222023	1.73383	2.94598E-06
Hydroxycinnamic acid derivatives	Caffeic acid 3-O-glucuronide	3.584229391	1.2747	0.000603618
Hydroxycinnamic acid derivatives	2-O-Caffeoylglucarate	1.84501845	6.99479	0.016135717
Hydroxycinnamic acid derivatives		1.706484642	1.31776	0.006341281
Hydroxycinnamic acid derivatives	Pulverulentoside I; (1aS,1bS,2S,5aR,6S,6aS)-6-({3-O-Acetyl-6-deoxy-2-O-[(2E)-3-(4-methoxyphenyl)-2-propenoyl]-?-L-mannopyranosyl}oxy)-1a-(hydroxymethyl)-1a,1b,2,5a,6,6a-hexahydrooxireno[4,5]cyclopenta[1,2-c]pyran-2-y l ?-D-glucopyranoside	4.032258065	1.47643	0.019366615
Fatty aldehydes		0.663129973	1.59182	0.013938561
Glycosilated furans		0.276319425	1.61669	1.66637E-05
Coumarins	Hydroxycoumarin	0.272925764	1.64369	0.026785014
Sugar derivatives	Starch acetate	0.344352617	1.42202	4.47207E-05
Alkaloids		0.610873549	1.05203	0.000382028
Alkaloids		6.134969325	1.07938	0.049262472
Iridoid glycosides	4-Methoxyphenyl 4-O-(b-D-galactopyranosyl)-b-D-glucopyranoside	2.06185567	2.18755	0.021022507
Coumarins	Clausarinol	6.535947712	1.96214	0.033974084
Carotenoids	Keto-γ-carotene	2.2172949	2.50714	0.011806631
Terpenoids	beta-ionone	2.392344498	1.73744	0.000169981
Terpenoids	Monoterpene propanoate	2.028397566	2.97708	0.000224076
Terpenes	(-)-Menthylacetate	7.142857143	1.53045	0.005427483
Flavonoids	Naringenin derivative	2.808988764	1.19424	0.017813444
Ketones	nonane-4,6-dione	0.463821892	1.07901	0.00077772
Diphenyls		0.412031314	2.31115	0.010615167

Supplemental Table S3. Pm-0-interval putative genes in courgette

***Cucurbita pepo* sbsp. *pepo* predicted (Holdsworh et al., 2016)**	***Arabidopsis thaliana* related**	**Function**	**CuGenDB related (cucurbitgenomics.org/)**
peroxidase 10, LOC111803951	peroxidase superfamily protein, AT1G49570	Heme binding, response to oxidative stress	Cp4.1LG10g02630
mitogen-activated protein kinase kinase kinase YODA, LOC111801447	YDA, AT1G63700	MAP kinase kinase kinase activity, protein binding, stomatal development regulatory pathway	Cp4.1LG14g06270
misato homolog 1, LOC111803136	plasma membrane, misato segment II, myosin-like, tubulin/FtsZ protein, AT4G37190		Cp4.1LG10g02770
transcription factor HHO5-like, LOC111787002	Homeodomain-like superfamily protein HHO5, AT4G37180	DNA-binding transcription factor activity, protein binding	Cp4.1LG19g11390
probable disease resistance protein At5g66900	N REQUIREMENT GENE 1.1, NRG1, N-terminal Resistance to Powdery Mildew 8 (RPW8) domain gene, AT5G66900	ADP binding, ubiquitin binding, required for signal transduction of TNLs	Cp4.1LG11g09670
glycerol-3-phosphate acyltransferase, chloroplastic-like, ATS1, LOC111811599	ATS1, AT1G32940	serine-type endopeptidase activity	Cp4.1LG05g00180
uncharacterized protein, LOC111804035	DOF zinc finger protein 1, AT1G51700	DNA-binding transcription factor activity	Cp4.1LG10g02700
autophagy-related protein 101, LOC111804543	meiotically up-regulated protein, AT5G66930	Protein binding, autophagosome assembly, autophagy	Cp4.1LG10g02660
salicylic acid-binding protein 2-like, SABP2, LOC111781756	methyl esterase 1, AT2G23620	hydrolase activity, acting on ester bonds, methyl indole-3-acetate esterase activity, methyl jasmonate esterase activity, methyl salicylate esterase activity	Cp4.1LG19g11360
nuclear pore complex protein NUP58-like, LOC111782033	unnamed protein product, C24_LOCUS20460		Cp4.1LG19g11610
dof zinc finger protein DOF3.4-like, LOC111804324	Dof-type zinc finger DNA-binding family protein, AT5G66940	DNA binding	
cyclin-H1-1, LOC111782267	cyclin H;1, AT5G27620	cyclin-dependent protein serine/threonine kinase regulator activity, protein kinase activity	Cp4.1LG19g11620
uncharacterized protein, LOC111803649	Pyridoxal phosphate (PLP)-dependent transferases superfamily protein, AT2G23520		Cp4.1LG10g02650
extra-large guanine nucleotide-binding protein 1-like, LOC111803714	XLG1, AT2G19140	Transposable element gene	Cp4.1LG10g02720

Supplemental Table S4. Primer used in this study

Name	Sequence
CP-Efl1a-F	5’-CCCGGACATCGTGACTTTAT-3’
CP-Efl1a-R	5’-ACCAGCTTCAAAACCACCAG-3’
CP-PR1-F	5’-AAACTCGGTGAGAATTGGATG-3’
CP-PR1-R	5’-CTTTGGCCTATGATATTGCCAC-3’
CP-PAL-F	5’-CATGGACAACACTCGTCTGG-3’
CP-PAL-R	5’-TTTGAAGGTAGCCCGTTGTT-3’
CP-NPR1-F	5’-GCCCAAGTGTTCAGAACCAAA-3’
CP-NPR1-R	5’-TCCAGTTCTTCCGAGCATTTC-3’
CP-PR2-F	5’-TGGTTCACCCCTTCTTGCTA-3’
CP-PR2-R	5’-ACAAGAGTTGGCGATTGCAT-3’
CP-MYC2-F	5’-GATCTGATGATCCAACAAGCC-3’
CP-MYC2-R	5’-ACACAGTGAAGGAGACAAGG-3’
CP-VSP2-F	5’-CTGATGAGTTTGATAGCTGGG-3’
CP-VSP2-R	5’-AGCAAAACCACCTTGAATCC-3’
CP-ETR1-F	5’-CCATACGCATACGCGAAAAG-3’
CP-ETR1-R	5’-AGTATTAGCCCGTCCATGCC-3’
CP-MMK1-F	5’-CCAGGACAGAGAATTAC-3’
CP-MMK1-R	5’-TGCATGCTGCTCGAAATCAAA-3’
CP-WRKY29-F	5’-AGCAAATTTCAGCTACCAAGCA-3’
CP-WRKY29-R	5’-CCCATGAATCTGAGCATACG-3’
CP-YODA-F	5’-CAATATGTAAGTCTACCTCACTAG-3’
CP-YODA-R	5’-CAAGCTACACTTCCATATTCCTC-3’
CP-SABP2-F	5’-ACGAAGCTGAGCCTTCAAC-3’
CP-SABP2-R	5’-GACAAGTCTTGCATGAACAATG-3’
CP-GST-F	5’-GTACATCGACGAAACTTGGAC-3’
CP-GST-R	5’-TTCTTCATCACTCTTCGCAC-3’
CP-BAK1-F	5’-GGACAGAGAGCCTTCGATCT-3’
CP-BAK1-R	5’-CGCAAGGTCGGGATCAACTA-3’
CP-LOX2.1-F	5’-ACAAGCCAGAGAATGTGTTGT-3’
CP-LOX2.1-R	5’-TCATCAGCCCATGCTTGTTC-3’
CP-PDF1.2-F	5’-GCCATGCTCAATTTCCTGG-3’
CP-PDF1.2-R	5’-CGTTTCAAACACACGTAGTAAG-3’

Supplemental Table S5. *P*-values of the two-way ANOVA analyses

Dependent variable	Factors (Two way-Anova)	P-value	Dependent variable	Factors (Two way-Anova)	P-value
YDA	variety	<0.001	**stomatal area**	variety	<0.0001
	treatment	0.019		treatment	<0.0001
	variety x treatment	0.014		variety x treatment	<0.0001
MMK1	variety	0.003	**SA levels**	variety	<0.0001
	treatment	0.041		treatment	<0.0001
	variety x treatment	0.002		variety x treatment	<0.0001
WRKY29	variety	0.05	**H3K4me3/HeK27me3 ratio**	variety	0.0053
	treatment	0.04		treatment	0.05
	variety x treatment	0.03		variety x treatment	0.0135
NPR1	variety	0.05	**Callose area**	variety	<0.0001
	treatment	0.008		treatment	<0.0001
	variety x treatment	<0.001		variety x treatment	0.0002
PR1	variety	0.05	**Callose volume**	variety	<0.0001
	treatment	0.01		treatment	<0.0001
	variety x treatment	0.05		variety x treatment	<0.0001
PR2	variety	<0.001	**CO_2 accumulaiton**	variety	<0.0001
	treatment	0.049		treatment	0.0089
	variety x treatment	0.038		variety x treatment	0.0004
PAL	variety	0.05	**Transpiration rate**	variety	<0.0001
	treatment	<0.001		treatment	0.0022
	variety x treatment	0.001		variety x treatment	0.0015
GST	variety	<0.001	**Stomatal conductance**	variety	<0.0001
	treatment	<0.001		treatment	0.0044
	variety x treatment	0.001		variety x treatment	0.0138

MYC2	variety	0.05	**Intercellular CO_2 concentration**	variety	0.0032
	treatment	<0.001		treatment	0.0125
	variety x treatment	0.039		variety x treatment	0.0149
ETR1	variety	<0.0001			
	treatment	<0.0001			
	variety x treatment	0.003			
VSP2	variety	0.048			
	treatment	0.05			
	variety x treatment	<0.0001			
SABP2	variety	<0.0001			
	treatment	0.004			
	variety x treatment	0.05			
LOX2.1	variety	<0.0001			
	treatment	0.006			
	variety x treatment	0.032			
